# Irreversible Inhibition of Human Ornithine Aminotransferase: A Quantum Mechanics/Molecular Mechanics Study

**DOI:** 10.3390/ijms27209272

**Published:** 2026-10-19

**Authors:** Yixin Zhang, Xu Guan, Anbao Gong, Guoqiang Wang, Qingzhu Zhang, Yanwei Li, Wenxing Wang, Qiao Wang

**Affiliations:** 1 Environment Research Institute, Shandong University, Qingdao 266237, China; zhangyixin@mail.sdu.edu.cn (Y.Z.); lyw@sdu.edu.cn (Y.L.); wxwang@sdu.edu.cn (W.W.); qiao.wang@sdu.edu.cn (Q.W.); 2 State Environmental Protection Key Laboratory of Land and Sea Ecological Governance and Systematic Regulation, Shandong Academy for Environmental Planning, Jinan 250101, China; guanxu@sdaep.com (X.G.); gonganbao@sdaep.com (A.G.)

**Keywords:** human ornithine aminotransferase, fluorine elimination, quantum mechanics/molecular mechanics, covalent inhibition mechanism

## Abstract

Human ornithine aminotransferase (hOAT) is overexpressed in hepatocellular carcinoma (HCC) and participates in glutamine anabolic metabolism, making it a promising therapeutic target. Although (1S,3S)-3-amino-4-(hexafluoropropan-2-ylidenyl)-cyclopentane-1-carboxylic acid (named here as BCF_3_ after the (bis)trifluoromethyl feature) has been proposed as a potent inhibitor of hOAT, previous experimental studies have characterized an external aldimine intermediate and the static end-state adduct, but the elementary fluorine-elimination steps and their modulation by the active-site environment remain incompletely understood. Herein, molecular dynamics (MD) and quantum mechanics/molecular mechanics (QM/MM) calculations were employed to systematically explore the irreversible inhibitory mechanism mediated by external aldimine II—the key reaction intermediate of BCF_3_—against hOAT. Conserved active-site water molecules act as proton-shuttling bridges, and residue-dependent electrostatic effects control fluorine elimination. External aldimine II forms a stable covalent adduct with Lys292 and sequentially releases fluorine atoms from the –CF_2_ moiety via water-mediated F^α−^ and F^β−^ elimination, with feasible barriers of 10.8 and 16.0 kcal/mol, respectively. Negative overall reaction energies indicate that the elimination products are energetically favored in the system. Electrostatic and noncovalent interaction analyses suggest that Arg180 and Gly42 promote fluorine elimination whereas Phe177 suppresses it, providing an atomistic explanation for the superior inhibitory potency of the bis-trifluoromethyl scaffold. The atomistic inhibitory mechanism uncovered in this study provides a theoretical foundation for the future rational design of hOAT-targeted drugs for hepatocellular carcinoma therapy.

## 1. Introduction

Hepatocellular carcinoma (HCC) is a primary liver cancer and the third leading cause of cancer death worldwide [1,2,3,4,5]. The disease is highly malignant, radiotherapy-resistant, and refractory to conventional chemotherapy, and even the standard-of-care targeted agent sorafenib offers only limited benefit [6,7,8,9]. Despite the emergence of multiple new targeted and immunotherapeutic regimens in recent years, drug resistance and poor long-term survival remain major clinical obstacles, creating an urgent demand for alternative therapeutic strategies based on tumor-specific metabolic vulnerabilities. Mounting evidence indicates that overexpression of human ornithine aminotransferase (hOAT) is tightly associated with proliferation and progression of HCC tumor cells, making hOAT a promising metabolic target for HCC therapy [10,11].

**Figure 1:**
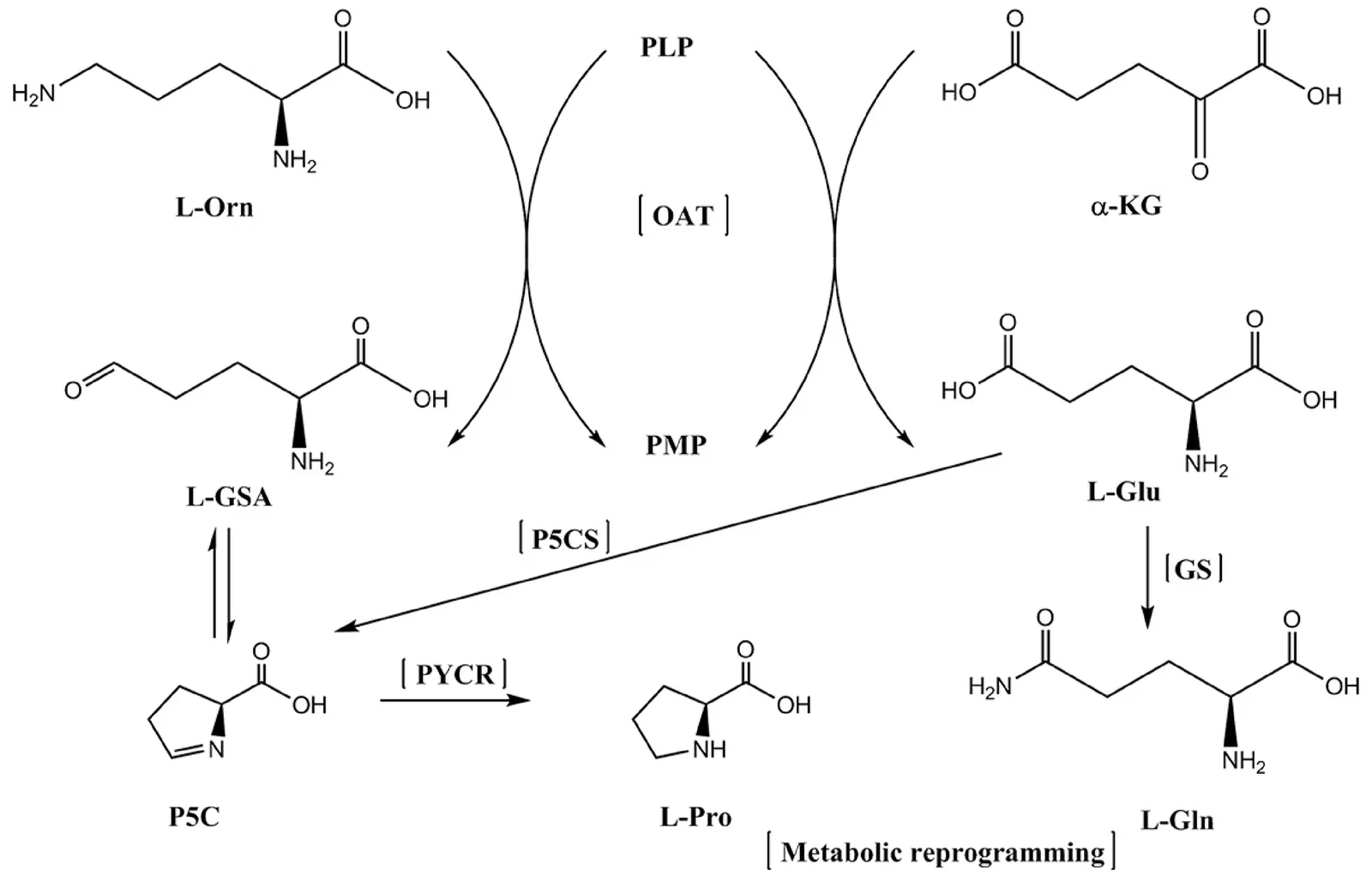
Chemistry of OAT.

Ornithine aminotransferase is a pyridoxal 5′-phosphate (PLP)-dependent enzyme found in the mitochondrial matrix of most human and animal tissues [12]. It participates in multiple core metabolic cascades and fulfills important detoxification functions in human physiology [13,14]. Mechanistically, two coupled half-reactions are involved in the transamination cycle of hOAT [15]. In the first half-reaction, L-ornithine is converted to L-glutamate-γ-semialdehyde with the formation of pyridoxamine phosphate intermediate. The latter is prone to cyclize to Δ1-pyrroline-5-carboxylate. In the second half-reaction, hOAT catalyzes the transfer of the amino group of pyridoxamine phosphate to α-ketoglutarate, forming glutamate and regenerating the PLP form of the cofactor. Moreover, L-Glu [16] is converted into L-Gln by glutamine synthetase (GS) [11], a process required by cancer cells to support glutamine-dependent anabolic processes and promote cellular proliferation (Figure 1). Consequently, hOAT has attracted considerable attention as a promising druggable target for developing novel anticancer interventions against HCC.

Preclinical in-vivo studies using HCC xenograft mouse models have validated the antitumor potential of mechanism-based hOAT inactivators [17]. Among the reported hOAT inactivators, (1S,3S)-3-Amino-4-(hexafluoropropan-2-ylidenyl)-cyclopentane-1-carboxylic acid (named here as BCF_3_ after the (bis)trifluoromethyl feature) is an irreversible inhibitor of hOAT that showed promising results for suppression of HCC tumor growth in athymic mice implanted with human-derived HCC [18]. Biochemical and crystallographic experiments have confirmed that BCF_3_ covalently modifies both the catalytic Lys292 residue and the PLP cofactor, generating a stable tethered ternary adduct that abolishes enzymatic activity. Quantitative kinetic analysis revealed that ~8% of BCF_3_ proceeds to irreversible covalent inactivation, and time-dependent ^19^F NMR detected fluoride ion accumulation under single-turnover conditions, directly supporting the fluorine-elimination step prior to PLP regeneration. Compared with monofluorinated or monotrifluoromethyl analogues, the bis-trifluoromethyl scaffold endows BCF_3_ with superior inhibitory potency, implying unique mechanistic features associated with successive fluorine-elimination events [16,18].

**Figure 2:**
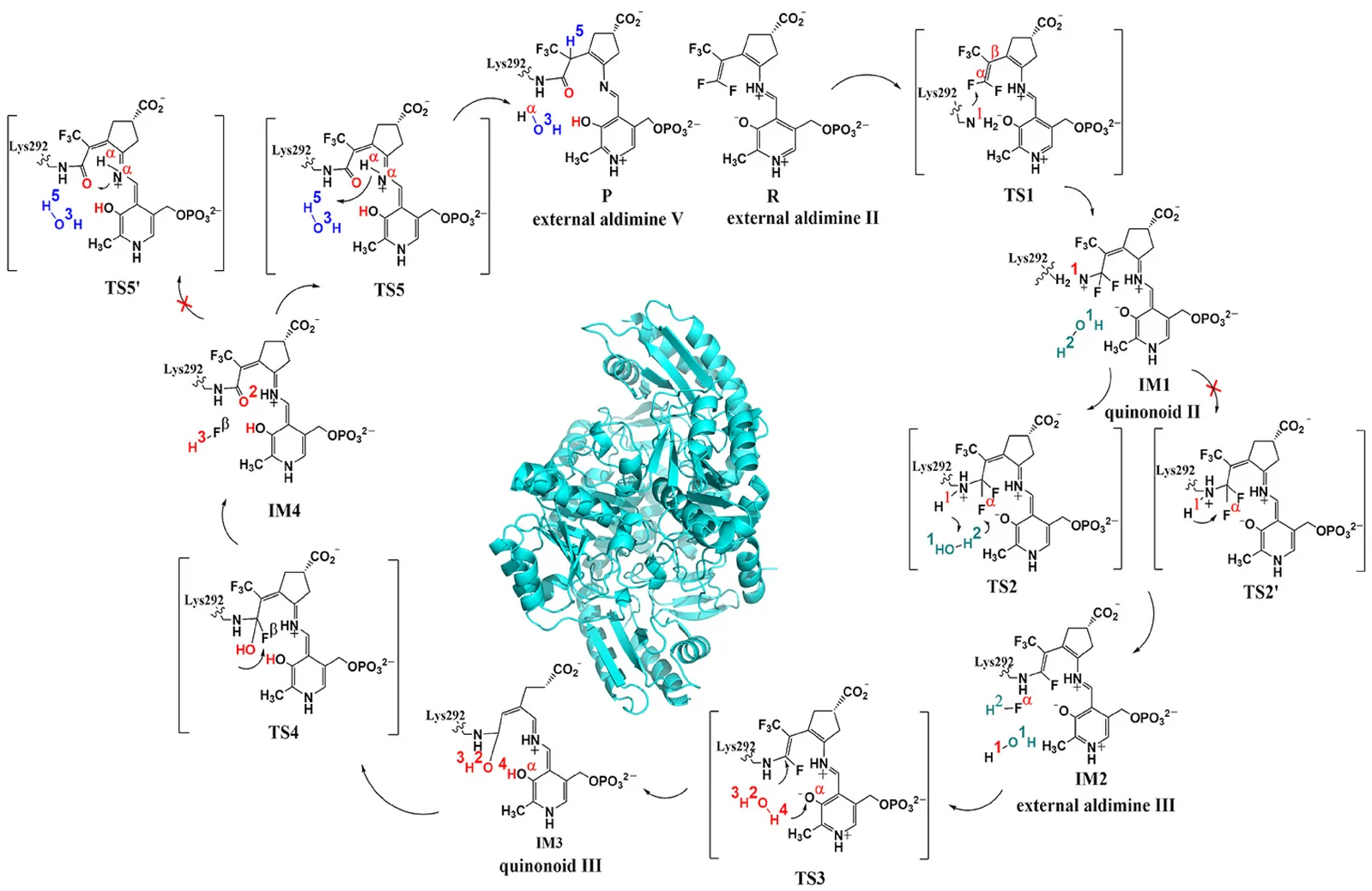
Proposed F^α−^ elimination and F^β−^ elimination mechanism of hOAT by external aldimine II.

Nevertheless, experimental techniques including X-ray crystallography and mass spectrometry only capture static end-state adduct information. Atomic-level mechanistic details describing the covalent inactivation cascade, especially the dynamic fluorine-elimination pathway, residue electrostatic modulation, and the functional role of active-site water molecules during inhibition, remain incompletely resolved [19]. To better characterize hOAT as a therapeutic target and accelerate the rational design of next-generation mechanism-based covalent inactivators for HCC treatment, it is imperative to dissect the full inhibitory mechanism at molecular resolution. Herein, we combine molecular dynamics (MD) simulations and quantum mechanics/molecular mechanics (QM/MM) calculations to unravel the atomistic-scale irreversible inhibition process triggered by the key external aldimine II intermediate (Figure 2). Our computational investigation provides mechanistic insights that will aid the rational design of covalent hOAT inhibitors in the near future.

## 2. Results and Discussion

The stability of the system can be evaluated by the root-mean-square deviation (RMSD), which is stable and maintains itself within 0.6 Å and 0.4 Å during the whole molecular dynamic process (Figure S1).

### 2.1. Molecular Dynamics of External Aldimine II Intermediate Within the hOAT Active Site

**Figure 3:**
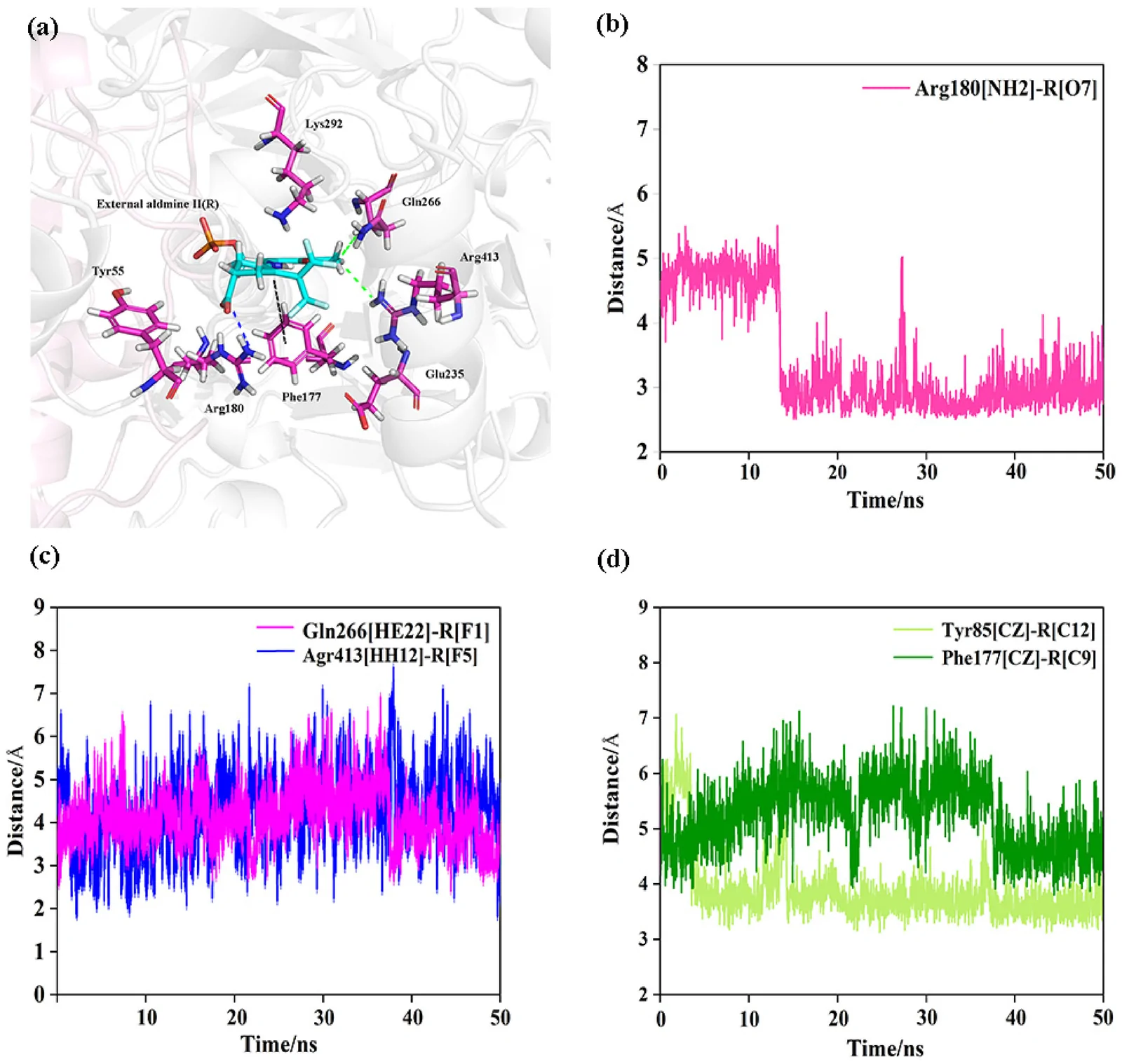
(**a**) Interaction of external aldimine II and crucial residue in active site; (**b**) the salt-bridge [N-O] distances from Arg180 with external aldimine II; (**c**) C–F···X contacts of external aldimine II with Gln266 and Arg413; and (**d**) hydrophobic [C-C] interactions of external aldimine II with Tyr85 and Phe177.

The binding mode of the external aldimine II intermediate within the hOAT active-site pocket is illustrated in Figure 3a. The blue, green and black dotted lines represent the salt-bridge, C–F···X and hydrophobic interactions, respectively [20,21]. The inhibitor carboxylate moiety forms ionic salt-bridge interactions with Arg180 during the inhibitory process [22]. Distance analyses derived from the 50 ns SBMD trajectories demonstrate that the salt-bridge established between the guanidinium nitrogen of Arg180 and the carboxylate oxygen of external aldimine II undergoes gradual convergence and becomes persistently stabilized at approximately 3 Å beyond 12 ns (Figure 3b). This observation underscores the critical role of this ionic contact as a dominant anchoring force that firmly locks the covalent intermediate within the active-site cavity.

Examination of intermolecular contacts for the bisfluoromethylenyl group reveals distinct dynamic behaviors for Gln266 and Arg413 in Figure 3c. Throughout the simulation, interatomic distances between fluorine atoms of the bistrifluoromethyl substituent and side-chain hydrogen atoms of Arg413 fluctuate predominantly within 4–5 Å, indicative of weak, transient non-bonded contacts. In contrast, distances between Gln266 and the bisfluoromethylenyl moiety undergo notable dynamic rearrangement, decreasing to the range of 3–4 Å after 35 ns simulation, pointing to enhanced close-range interactions in the later stage of MD simulation. Given the geometry and the electron-withdrawing environment of the –CF_2_ moiety, these close contacts are best described as weak C–F···H–N/O interactions rather than halogen bonding. Given the geometry and the strongly electron-withdrawing environment of the –CF_2_ moiety, these close contacts are better described as weak C–F···H–N/O interactions rather than conventional halogen bonding. It is therefore speculated that Gln266 may stabilize the bis(trifluoromethyl) moiety through these enhanced close-range interactions during the later stage of the simulation.

As shown in Figure 3d, hydrophobic residues Tyr85 and Phe177 participate in substrate positioning via hydrophobic packing effects. Trajectory-based distance statistics reveal that heavy-atom separations between Tyr85 and the inhibitor remain stably maintained near 4 Å for the majority of the simulation timescale. Such close proximity raises the possibility of weak CH-π contributions between the aliphatic moiety of external aldimine II and the aromatic ring of Tyr85, alongside conventional hydrophobic van der Waals contacts. In contrast, the hydrophobic interaction involving Phe177 exhibits pronounced temporal evolution: the inter-residue distance starts from a relatively large value and only converges to 5 Å after 35 ns of simulation. This larger interatomic separation suggests that Phe177-mediated contacts are dominated by generic hydrophobic packing, with negligible CH-π character. This delayed compaction implies that the bulky aromatic side-chain of Phe177 progressively occupies partial active-site volume in the later simulation phase, which mechanistically corroborates its sterically inhibitory influence on the fluorine-elimination reaction revealed by subsequent electrostatic decomposition analysis. Collectively, the synergistic contributions from electrostatic, ionic and hydrophobic interactions shape the geometry of the active-site cavity to accommodate external aldimine II.

Consistent with prior experimental reports, the bistrifluoromethyl-substituted inhibitor exhibits superior inhibitory potency compared with its monotrifluoromethyl counterpart [17,18,22]. On the basis of both static structural insights and dynamic MD characterizations, the improved inhibitory efficacy conferred by the bistrifluoromethyl moiety can be rationalized by two key aspects: this substituent enables more favorable steric filling of the hOAT active-site pocket, while the additional trifluoromethyl group furnishes supplementary weak non-covalent contacts to reinforce inhibitor-enzyme association.

### 2.2. Individual Residue Influence

**Figure 4:**
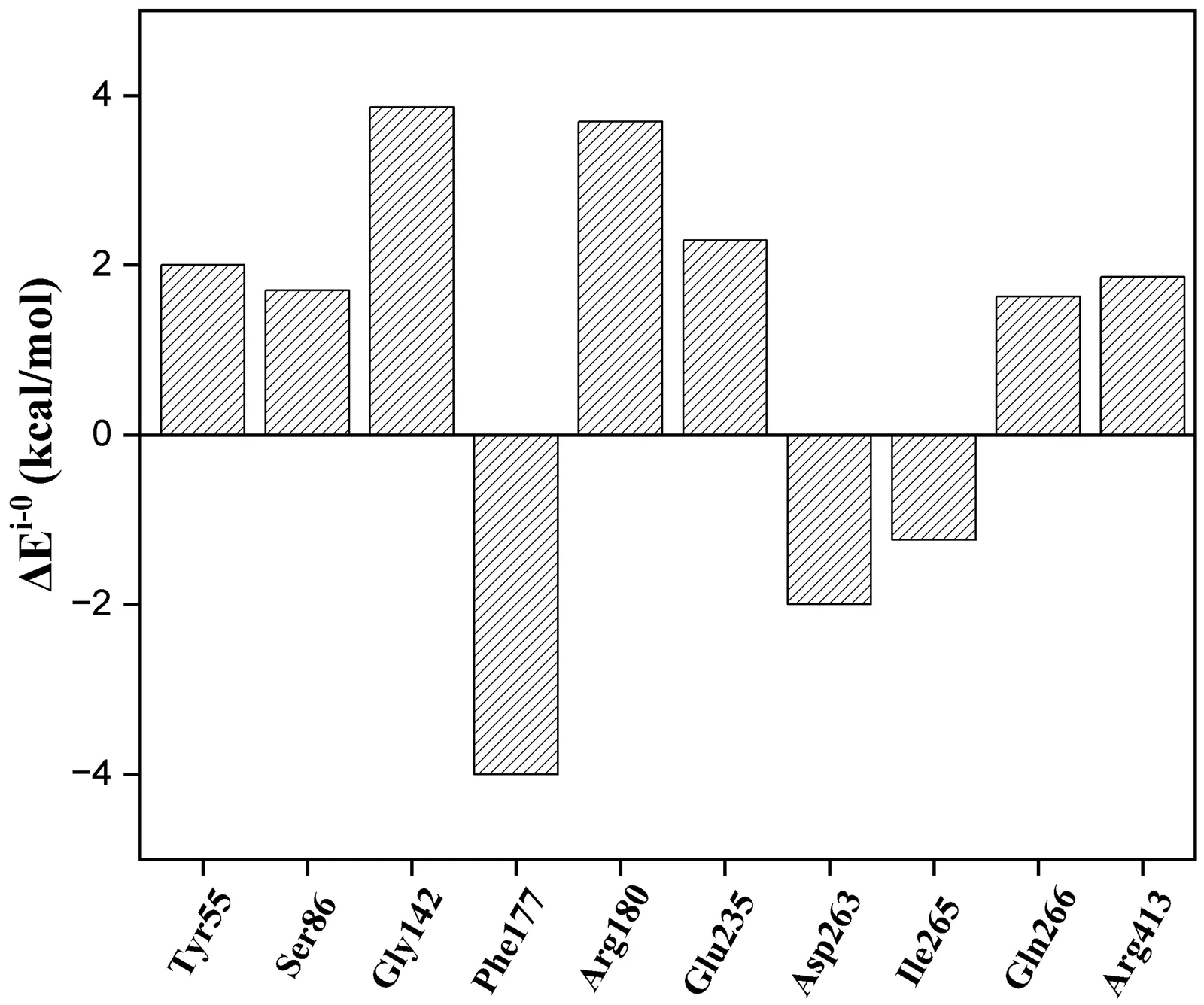
Δ*E*^*i*−^^0^ values of 10 individual residues toward the fluorines elimination reaction. The amino acids are calculated to research the electrostatic influence.

Considering that the fluorine-elimination reaction of external aldimine II (R) may be electrostatically affected by the surrounding residues, we performed electrostatic analysis of ten amino acid residues during the reaction. The energy contribution is quantified by the following formula:

Δ*E^i^*^−0^ = Δ*E^i^* − Δ*E*^0^

where Δ*E^i^* is the energy with charges on residue *i* set to 0, Δ*E*^0^ is the original energy barrier value, and Δ*E^i^*^−0^ is the energy change considering the electrostatic effect of residue *i*. The positive value of Δ*E^i^*^−0^ indicates that the residue promotes the fluorines elimination reaction, otherwise it inhibits the reaction. As illustrated in Figure 4, seven residues—Tyr55, Ser86, Gly42, Arg180, Glu235, Gln266 and Arg413—yield positive Δ*E^i^*^−0^ values (|Δ*E^i^*^−0^| > 1 kcal/mol), pointing to their electrostatic facilitation toward fluorine elimination. Among these promoting residues, Gly42 and Arg180 exhibit the largest positive Δ*E^i^*^−0^ magnitudes. The positively charged guanidinium group of Arg180 can stabilize the accumulating negative charge on fluorine atoms during C-F bond cleavage, which is also consistent with our MD observation that Arg180 forms persistent salt-bridge anchoring with the carboxylate moiety of external aldimine II. The polar residues Tyr55, Ser86, Gln266 and positively charged Arg413 provide moderate electrostatic promotion. By contrast, Phe177, Asp263 and Ile265 display negative Δ*E^i^*^−0^ values, indicative of electrostatic suppression on the elimination reaction. Phe177 possesses the most negative Δ*E^i^*^−0^ value among all tested residues, which may reduce the active space and be unconducive to the reaction.

### 2.3. Irreversible Inhibition and F^α−^ Elimination

**Figure 5:**
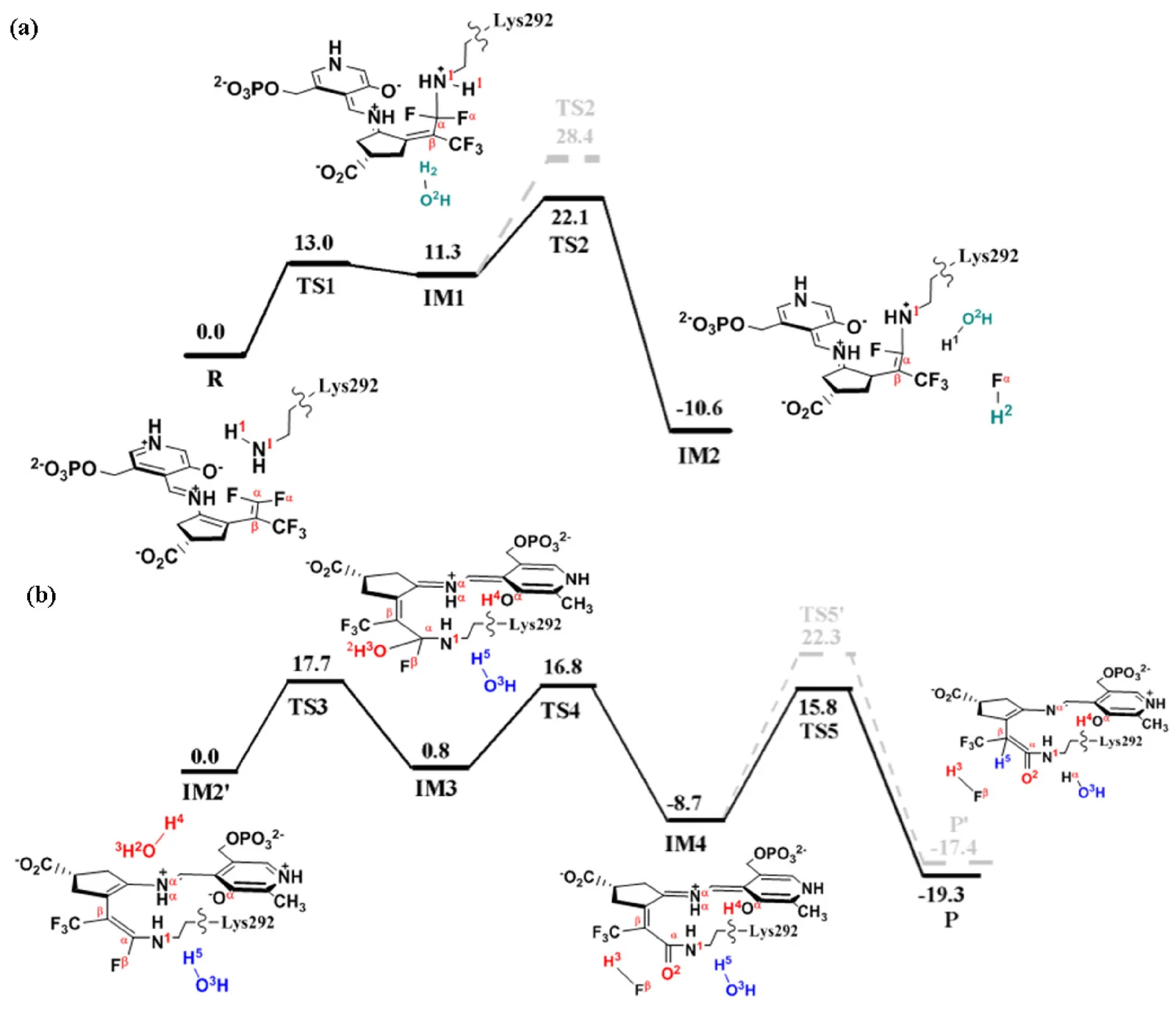
Boltzmann-weighted average energy profile of four snapshots in (**a**) F^α−^ elimination mechanism and (**b**) F^β−^ elimination mechanism of the QM region.

The irreversible inhibition mechanism of hOAT is shown in Figure 2. Initially, the side-chain amino group of Lys292 attacks the electrophilic bisfluoromethylenyl moiety of external aldimine II (R) to yield the quinonoid II intermediate (IM1), with a Boltzmann-weighted average energy barrier of 13.0 kcal/mol (Figure 5a). Inspection of the optimized reactant structure (Figure S4a) shows that the distance between H^1^ (from Lys292) and F^α−^ is 2.51 Å, a geometric feature that naively appears amenable for direct proton transfer and potential hydrogen-fluorine interaction. Nevertheless, this direct proton-transfer pathway yields a prohibitively high energy barrier of 28.0 kcal/mol, which renders this channel kinetically inaccessible under physiological conditions (Structures and key bond distances are provided in Figure S6).

**Figure 6:**
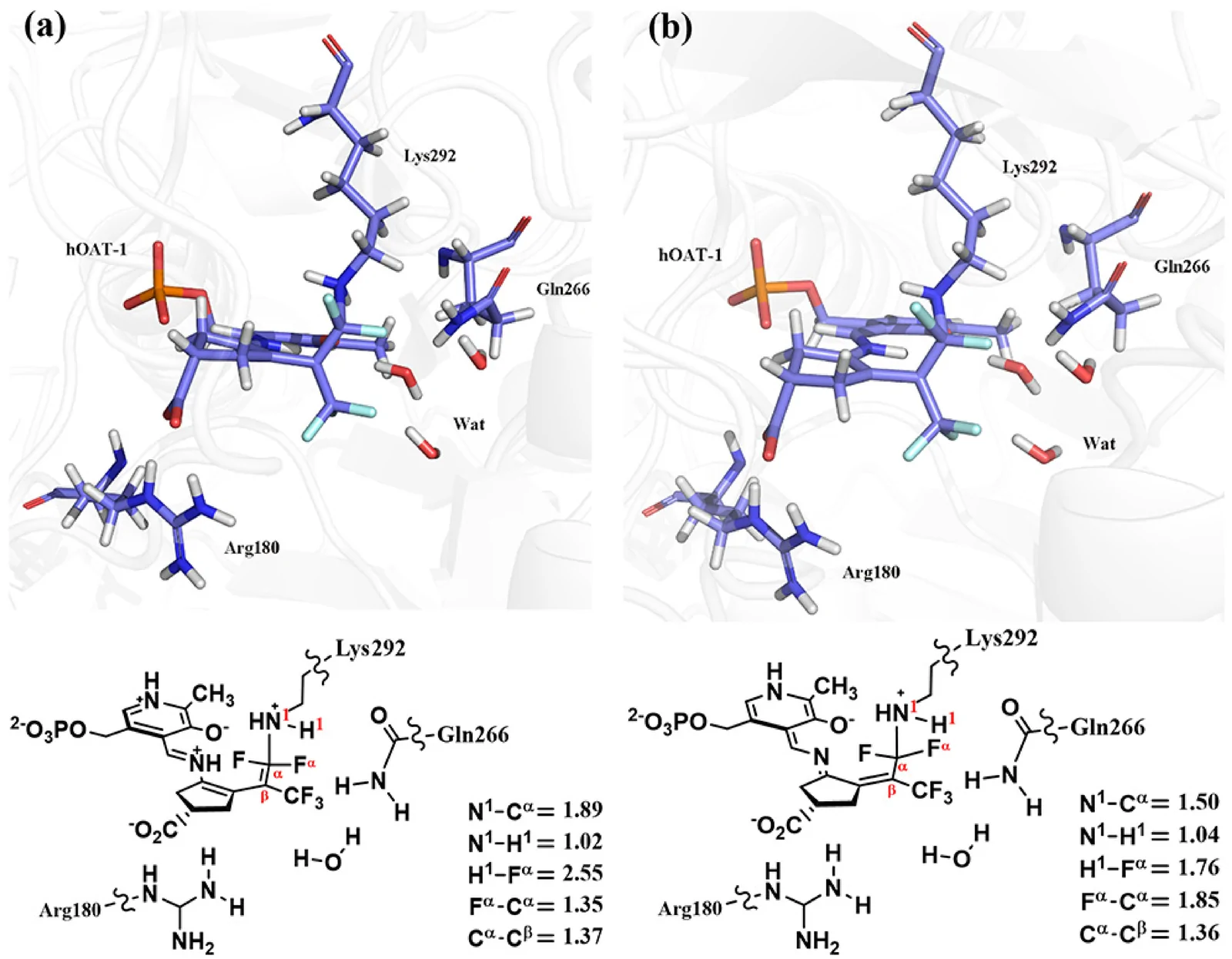
Optimized structures and key bond distances (32.56 ns) for the reactant (**a**) TS1 and (**b**) TS2.

Significantly, one conserved water molecule residing within the active-site cavity participates in the catalytic cycle and functions as a “proton-transfer bridge” between Lys292 and the F^α^ atom of the inhibitor (Figure 6a). To verify the positional stability of this catalytic water, a water occupancy analysis was performed over the post-equilibration trajectory using the VolMap plugin in VMD (Supporting Information, Figure S3). The analysis reveals a persistent high-occupancy region near Lys292 and the −CF_2_ moiety, indicating that although individual water molecules are exchanged, the catalytic hydration site remains continuously occupied throughout the simulation. Consistent with this, at the TS2 transition-state geometry, the H^1^–O^1^ distance reaches 3.39 Å, and the H^2^–F^α^ distance shortens to 2.01 Å. These key interatomic distances reflect a concerted proton-shuttling character: proton H^1^ partially departs from the amine group of Lys292 toward the bridging water oxygen, while proton H^2^ from the water molecule partially migrates toward the leaving F^α^ atom. In addition, F^α−^ elimination is highly exergonic, providing considerable thermodynamic driving force for the forward direction. Together with fluoride dissociation into bulk solvent, this renders the reaction kinetically irreversible, consistent with experimental mechanistic hypotheses. By mediating proton relay through the bridging water molecule, the system circumvents the energetically demanding direct proton transfer from Lys292 to fluorine. This water intermediate delocalizes the unfavorable charge redistribution that would otherwise accumulate on Lys292 and the departing fluoride in the water-free pathway, lowering the Boltzmann-weighted average barrier to 10.8 kcal/mol (Method S1), which is kinetically accessible for enzymatic catalysis. The F^α^ elimination step is exothermic, thermodynamically driving product formation and confirming its irreversible character. This result corroborates prior experimental mechanistic hypotheses for hOAT inactivation by fluorinated mechanism-based inhibitors.

### 2.4. Subsequent F^β−^ Elimination Mechanism

Following F^α−^ elimination, additional fluorine atoms belonging to the fluoromethylenyl moiety can undergo further elimination, which reinforces the covalent association between the inhibitor and hOAT (Figure 2). Analogous to the F^α−^ elimination step, a water molecule continues to serve a critical functional role throughout the F^β−^ elimination cascade. This water molecule resides in the interstitial space between the bisfluoromethylenyl substituent and the PLP cofactor. At the reactant-like geometry shown in Figure S5a, the H^4^-O^α^ distance is 1.88 Å and the O^1^–C^α^ distance measures 3.16 Å. Such a geometric arrangement facilitates the progression of subsequent chemical transformations and sets up hydrogen-bonding interactions between H^3^ and F^β^.

**Figure 7:**
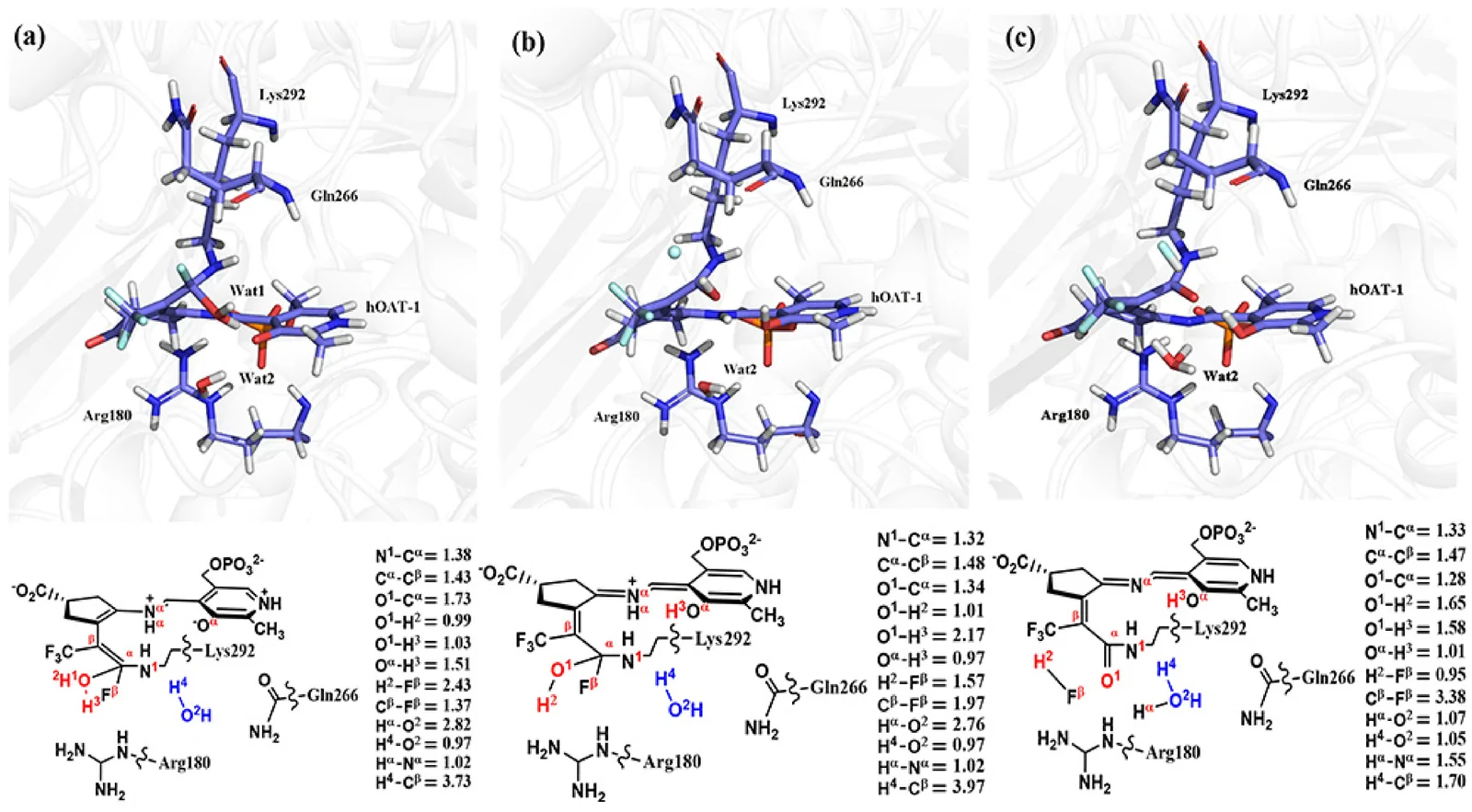
Optimized structures and key bond distances (32.56 ns) for the reactant (**a**) TS3, (**b**) TS4, and (**c**) TS5.

In the ensuing step, the water-derived O^2^ atom attacks the C^α^ carbanion, while the hydroxyl group of PLP establishes hydrogen-bonding contacts with the bridging water molecule. In TS3, the H^4^–O^α^ distance shortens to 1.51 Å (Figure 7a), corresponding to a Boltzmann-weighted average energy barrier of 17.7 kcal/mol. Cleavage of the O^2^–H^3^ bond then takes place to generate hydrogen fluoride and a ketone-containing intermediate, with a Boltzmann-weighted average barrier of 16.0 kcal/mol. In the final elementary step, the water molecule again operates as a proton-transfer bridge, shuttling the imino-derived H^α^ proton toward the double-bonded C^β^ carbon. This step exhibits the highest Boltzmann-weighted average barrier of 24.5 kcal/mol and thus represents the rate-determining step for the entire F^β−^ elimination sequence (Structures of TS5′ are provided in Figure S7). The overall reaction energy for F^β−^ elimination reaches −19.3 kcal/mol (from reactant IM2′ to final product P), demonstrating strong thermodynamic favorability for the forward multistep transformation.

### 2.5. Electrostatic Potential and Noncovalent Interaction

**Figure 8:**
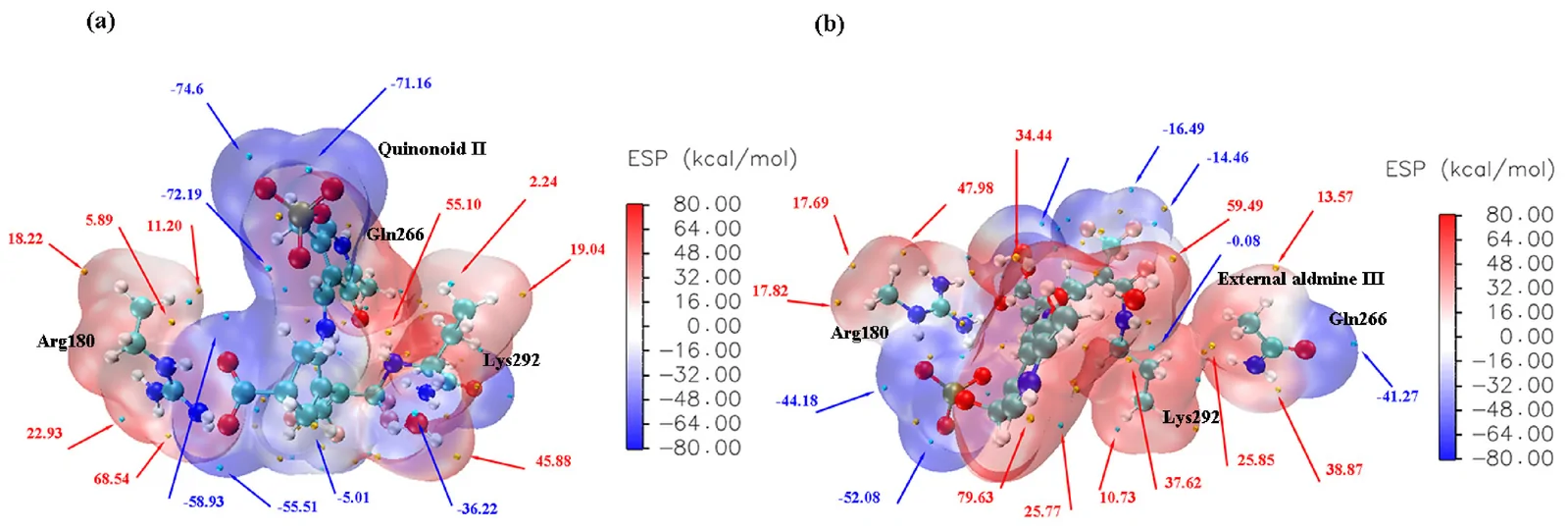
ESP on the molecular vdW surface of the reactant. The unit is in kcal/mol. Surface local maxima and minima of ESP values are presented as red and blue spheres. Species shown: (**a**) IM1; (**b**) IM2.

To gain deeper mechanistic insight into the fluorine-elimination pathway, electrostatic potential (ESP) and non-covalent interaction (NCI) analyses were performed. The molecular van der Waals surface ESP maps together with surface extrema for intermediates IM2 and IM3 are presented in Figure 8. For IM2, the amino-group moiety of Lys292 exhibits a positive ESP value of 55.10 kcal/mol, whereas the adjacent catalytic water molecule shows moderately negative ESP (−36.22 kcal/mol). This complementary electrostatic signature indicates that the nitrogen site N^1^ acts as a reactive center and favors proton-shuttling events between Lys292-derived N^1^ and the water oxygen O^1^. Furthermore, positively valued ESP regions of IM2 (Figure 8b) cover the amino group, alkyl framework, and nearby catalytic water. Hydrogen atoms belonging to this water molecule display a prominent positive character, reaching a global surface ESP maximum of +59.49 kcal/mol. These ESP observations support our proposed reaction picture, in which the catalytic water participates in sequential proton transfer during fluorine elimination.

**Figure 9:**
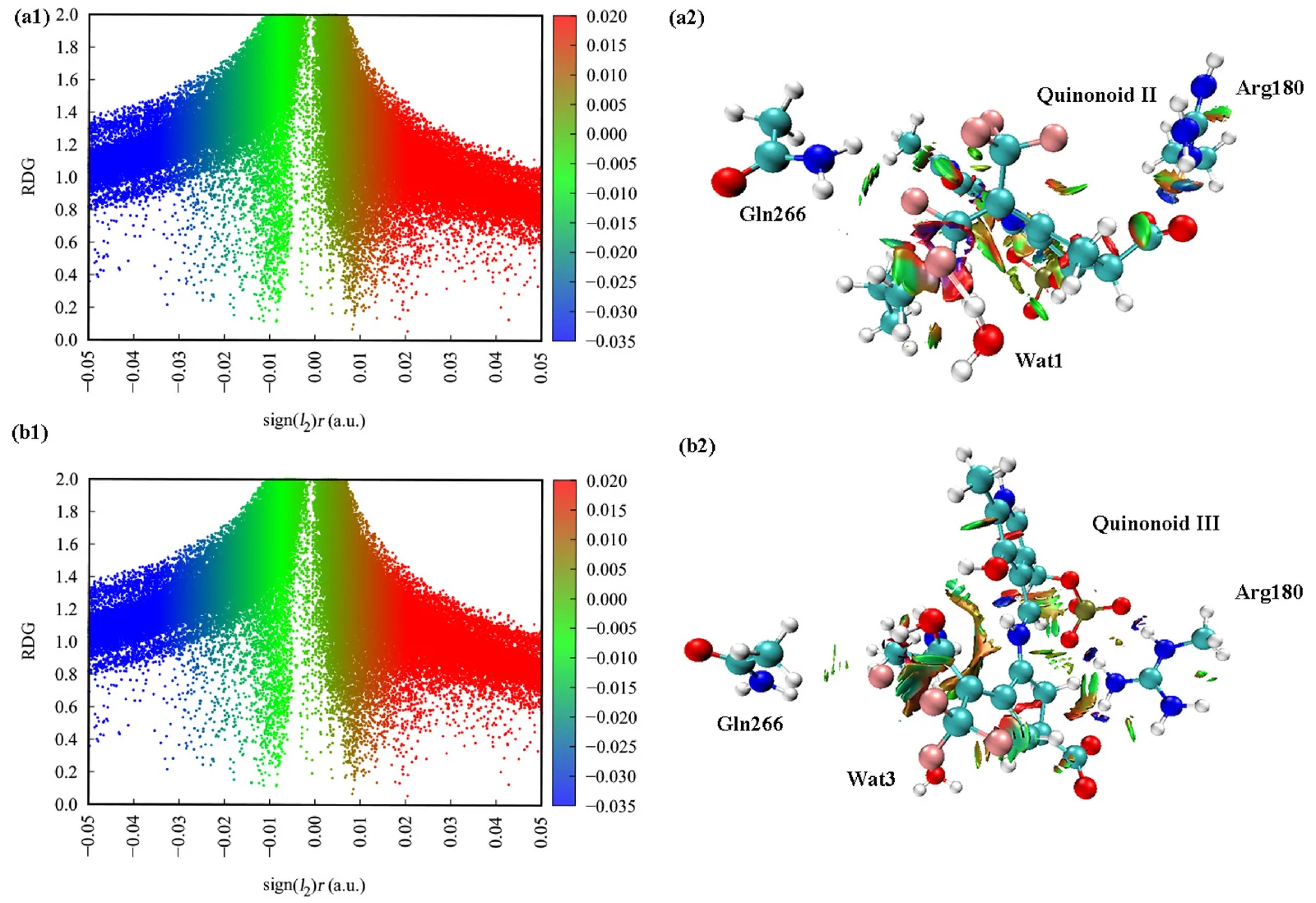
Color-mapped reduced density gradient (RDG) isosurface graphs and scatter diagrams of external aldimine II and three critical amino acids. Species shown: (**a1**,**a2**) TS2; (**b1**,**b2**) TS4.

Figure 9 shows NCI scatter plots characterizing weak intermolecular contacts between external aldimine II and key active-site residues at transition states TS2 and TS4. The horizontal axis corresponds to the ρ(r)sign(λ2) function ranging from −0.05 to 0.05 a.u., while the vertical axis denotes the reduced-density-gradient (RDG) function spanning 0–2 a.u. A unified color scale from −0.035 to 0.020 a.u. was adopted for all NCI analyses: blue regions denote strong attractive interactions (e.g., hydrogen bonds and halogen bonds), green corresponds to weak van der Waals dispersion contacts, and red signifies strong steric repulsion.

Distinct strong hydrogen-bonding interactions can be identified between quinonoid II and residues Gln266 and Arg180 at TS2. Amino-group hydrogens form favorable hydrogen-bond networks with the bridging catalytic water, which stabilizes the transition-state geometry and facilitates fluorine elimination. Comparable non-covalent features are observed for quinonoid III at TS4, where the water molecule incorporated into the substrate establishes hydrogen-bond contacts with the O^α^ site and fluorine substituents. These stabilizing hydrogen-bond interactions manifest as characteristic spikes falling within −0.03 to 0.01 a.u. on the NCI scatter plots and correspond to green-blue NCI isosurfaces. Collectively, both ESP and NCI analyses rationalize the essential role of the catalytic water and key polar residues in lowering the reaction barrier for sequential fluorine-elimination steps.

## 3. Materials and Methods

### 3.1. Molecular Dynamic Simulations

The initial structure was constructed based on the X-ray structure of the hOAT in complex with BCF_3_ (PDB code: 6OIA, resolution: 1.8 Å) [23]. For the first stage of F^α−^ elimination, the original substrate from the crystal structure was manually modified to yield external aldimine II. The protonation state of amino acid residues under the experimental condition pH = 7.4 was determined [23,24]. The CHARMM force field for substrate was generated by SwissParam (Swiss Institute of Bioinformatics, Lausanne, Switzerland) [25]. The missing hydrogen atoms in the system were added and optimized using the HBUILD module of CHARMM (version 42b2; Harvard University, Cambridge, MA, USA). The system was dissolved in a TIP3P water sphere model with a diameter of 70.0 Å [26]. Six potassium ions were randomly added to the system to maintain its neutrality, and the whole system comprised 2099 atoms. The temperature of the system was increased from 0 K to 298.15 K within 50 ps, and the thermodynamic equilibrium state was reached at approximately 500 ps with a 1 fs time step. Then, 50 ns stochastic boundary molecular dynamics (SBMD) was performed under the NVT ensemble at 298.15K. The full system was allowed to move freely, with no restraints applied throughout the simulation. The leap-frog algorithm and Langevin dynamics method in the CHARMM package were implemented in the simulation process. Thousands of frames were generated in the MD simulations, and representative active-site conformations were extracted from the post-equilibration trajectory using TT-Cluster (Trusty Trajectory Clustering, version 4.6; Institute for Integrative Biology of the Cell (I2BC), CEA, CNRS, Université Paris-Saclay, Gif-sur-Yvette, France) analysis for subsequent QM/MM calculations (Figure S2). An analogous procedure was applied for the second stage of F^β−^ elimination; full details are provided in the Supporting Information.

### 3.2. QM/MM Methodology

The ChemShell 3.7.1 platform (Science and Technology Facilities Council, Daresbury Laboratory, Warrington, UK) [27], coupled with the quantum chemistry program Turbomole 7.2 (TURBOMOLE GmbH, Karlsruhe, Germany) [28] and the molecular mechanic module DL-POLY (module revision 3.7.1) [29], was employed to calculate the QM and MM regions, respectively. The interface between the QM and MM regions was handled using the charge-shift link-atom scheme [30]. The QM region of F^α−^ elimination stage (87 atoms) contains the substrate external aldimine II, Lys292, Gln266, Arg180 and a water molecule. The M06-2x/def2-SVP//CHARMM27 level of theory was used for geometry optimization, whereas the M06-2x/def2-TZVP//CHARMM27 level was used for the single point energy calculation. More details about the F^β−^ elimination stage are provided in the Supporting Information.

### 3.3. Real Space Functions

Quantitative electrostatic potential (ESP) analysis was performed to determine the possibility of reaction. This approach uses the maxima and minima of ESP values mapped onto the van der Waals (vdW) surface to identify putative reaction sites [31]. Positive ESP values arise from nuclear positive charge, while negative values originate from electron density. Larger absolute ESP magnitudes indicate stronger local intermolecular interaction potential and can modulate neighboring non-covalent contacts [32,33].

The non-covalent interaction (NCI) analysis relies on the electron density in real space and the reduced gradient density function (RDG) [34]. The value of ρ(r)sign (λ2) is projected in different colors on the RDG isosurface. ρ(r) is the threshold point for measuring the strength of interaction in atoms in molecules (AIM) theory. It is combined with the second eigenvalue sign (λ2) of the electron density hessian matrix to evaluate the type of interaction [35]. Multiwfn 3.72 (Beijing Kein Research Center for Natural Sciences, Beijing, China) [36] was used for wave function analysis, while color-filled RDG isosurfaces and scatter plots were drawn via VMD 1.9.3 (Theoretical and Computational Biophysics Group, University of Illinois Urbana-Champaign, Urbana, IL, USA) [37] and Gnuplot program (version 5.4, Gnuplot Development Team) [33]. When visualized in molecular-analysis software, these outputs directly reveal the spatial location, magnitude, and nature of weak intermolecular interactions. In this paper, blue, green, and red color correspond to hydrogen-bond or halogen-bond-type interaction, van der Waals (vdW) interaction and steric hindrance, respectively.

## 4. Conclusions

Leveraged to MD simulations and QM/MM calculations, the present work systematically elucidates the atomic-level irreversible inhibitory mechanism of the key external aldimine II intermediate of BCF_3_ against hOAT. The covalent trapping of hOAT is initiated by stable covalent linkage between external aldimine II and the catalytic Lys292 residue, followed by sequential dual fluorine elimination cascades, namely, F^α−^ and F^β−^ elimination, which collectively underpin the irreversible inactivation of hOAT. The F^α−^ elimination pathway proceeds via two elementary steps with an overall exothermic reaction energy of −10.6 kcal/mol, while the subsequent F^β−^ elimination process involves three successive reaction steps and yields a more favorable energetic change of −29.9 kcal/mol relative to the initial external aldimine II reactant (R). The substantially negative overall reaction energies indicate strong thermodynamic spontaneity for both elimination processes. Combined with fluoride release into bulk solvent, these reactions exhibit kinetic irreversibility, accounting for robust long-term hOAT inactivation induced by the bis-trifluoromethyl inhibitor. Electrostatic decomposition analyses reveal that Arg180 and Gly42 prominently promote fluorine elimination, whereas Phe177 exerts a dominant inhibitory effect. Combined ESP and NCI results further verify that residue-derived hydrogen-bond networks and noncovalent interactions stabilize transition-state geometries and facilitate inhibitor binding. This work systematically clarifies the dynamic binding features, water-assisted catalytic pathway, and residue electrostatic modulation underlying BCF_3_-mediated hOAT inactivation. The obtained atomistic mechanistic insights provide reliable guidance for the future rational design of improved hOAT-targeted covalent inhibitors against HCC.

## Data Availability

The original contributions presented in this study are included in the article/Supplementary Materials. Further inquiries can be directed to the corresponding authors.

## Supplementary Materials

The following supporting information can be downloaded at https://www.mdpi.com/article/10.3390/ijms27209272/s1. References [38,39] are cited in the supplementary materials (Method S1).
