# Supplementary material for: Irreversible Inhibition of Human Ornithine Aminotransferase: A Quantum Mechanics/Molecular Mechanics Study
Source: Int J Mol Sci. 2026 Oct 19;27(20):9272. doi: 10.3390/ijms27209272 (PMC13705100; doi:10.3390/ijms27209272)
Supplement: Supplementary file 1 [file ijms-27-09272-s001.zip › ijms-4573133-supplementary.pdf]

Table of Contents

Theoretical Details.

Method S1. Boltzmann weighted average method.

Figure S1. The curve of distance RMSD with time in two molecular dynamics simulation (50 ns).

Figure S2. TT-Clust applied to the molecular dynamics simulation made with CHARMM. (a) Cluster dendrogram of the three clusters. (b) Cluster analysis of the system along the trajectory. (c) The RMSD between each conformation and all conformations in molecular dynamics simulation. (d) 2D projection of the distance (RMSD) between the representative frame of each cluster.

Figure S3. Fractional-occupancy volumetric map for water oxygen atoms from equilibrated 50 ns SBMD simulation.

Figure S4. Optimized structures and key bond distances (32.56 ns) for the reactant (a) R, (b) IM1, (c) IM2.

Figure S5. Optimized structures and key bond distances (32.56 ns) for the reactant (a) IM2', (b) IM3, (c) IM4, (d) P.

Figure S6. Structures and key bond distances of unoptimized pathway (32.56 ns) for the reactant (a) TS2', (b) IM2.

Figure S7. Structures and key bond distances of unoptimized pathway (32.56 ns) for the reactant (a) TS5', (b) P'.

The force field of external aldimine II and Lss generated by SwissParam.

1 Molecular Dynamic Simulations

In the second stage of F^{β} elimination, the protonation state of amino acid residues under the experimental condition pH = 7.4 was determined. CHARMM force field for substrate was generated by SwissParam. The missing hydrogen atoms in the system were added and optimized using the HBUILD module of CHARMM. The system was dissolved in a TIP3P water sphere model with a diameter of 70.0 Å. Seven potassium ions were randomly added to the system to maintain its neutrality, and the whole system contains 2276 atoms. The temperature of the system was increased from 0 K to 298.15 K within 50 ps, and the thermodynamic equilibrium state was reached at approximately 500 ps with a 1 fs time step. Then, a 50 ns stochastic boundary molecular dynamics (SBMD) was performed under the NVT ensemble at 298.15K, and the entire system was allowed to move freely during the simulation No restrains were used during the simulation. The leap-frog algorithm and Langevin dynamics method in the CHARMM package were implemented in the simulation process. Thousands of frames were generated in molecular dynamics (MD) simulations, from which stable structures were randomly selected for QM/MM calculations.

2 QM/MM Methodology

The ChemShell 3.6.0 platform, coupled with the quantum chemistry program Turbomole 7.2 and the molecular mechanic module DL-POLY, was employed to calculate the QM and MM regions respectively. The boundary between the QM region and the MM region was processed by a charge shift model connecting hydrogen atoms. The QM region of F^{β} elimination stage (88 atoms) contains the substrate external aldimine II, Lss292, Gln266, Arg180 and two water molecules. The M06-2x/def2-SVP//CHARMM27 level was implemented for structure optimization, and M06-2x/def2-TZVP//CHARMM27 level was used for the single point energy calculation.

Method S1. Boltzmann weighted average method.

Four representative conformations from each stage were extracted from the trajectory for su bsequent QM/MM calculations. The rate constant of an enzyme-catalyzed reaction generally exhi bits a wide range of fluctuations. It is assumed that the conformations obtained from the trajector y of the molecular dynamic simulations correspond to the local rate constants [1, 2]. Thus, the Q M/MM energies are calculated and employed to obtain average barrier via the formula of Boltzm ann weighted average method. In this formula, R is the gas constant, n is the number of selected s napshots, T is the temperature (K), ΔEi is the energy barrier of snapshot i and ΔE is the average e nergy barrier.

$$\Delta E = -RT \ln\left\{\frac{1}{n}\sum_{i=1}^{n} \exp\left(\frac{-\Delta E_i}{RT}\right)\right\}$$

[1]Cooper, A.M., Kaestner, J., 2014. Averaging Techniques for Reaction Barriers in QM/MM Simulations. Chemphyschem 15(15), 3264-3269. https://doi.org/10.1002/cphc.201402382.

[2]Li, Y., Zhang, R., Du, L., Zhang, Q., Wang, W., 2016. How Many Conformations of Enzymes Should Be Sampled for DFT/MM Calculations? A Case Study of Fluoroacetate Dehalogenase. International Journal of Molecular Sciences 17(8), 1372. https://doi.org/10.3390/ijms17081372.

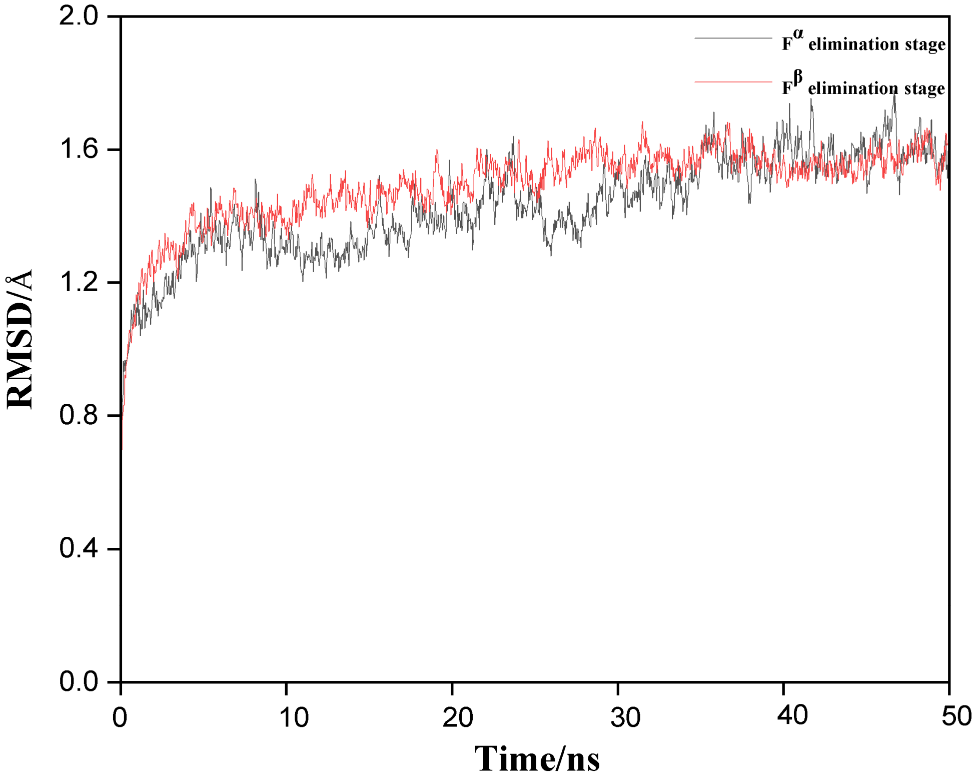

Figure S1. The curve of distance RMSD with time in two molecular dynamics simulation (50 ns).

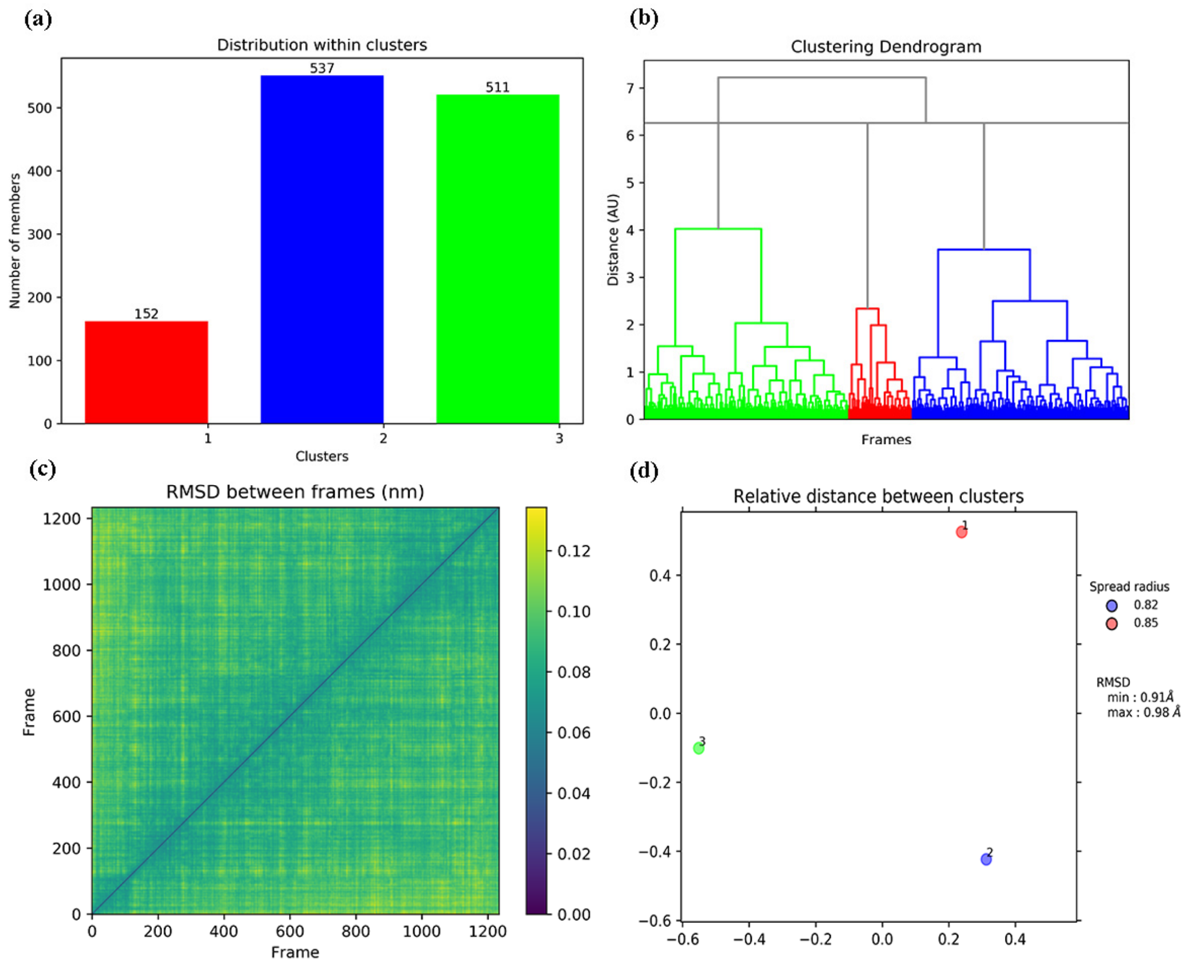

Figure S2. TT-Clust applied to the molecular dynamics simulation made with CHARMM. (a) Cluster dendrogram of the three clusters. (b) Cluster analysis of the system along the trajectory. (c) The RMSD between each conformation and all conformations in molecular dynamics simulation. (d) 2D projection of the distance (RMSD) between the representative frame of each cluster.

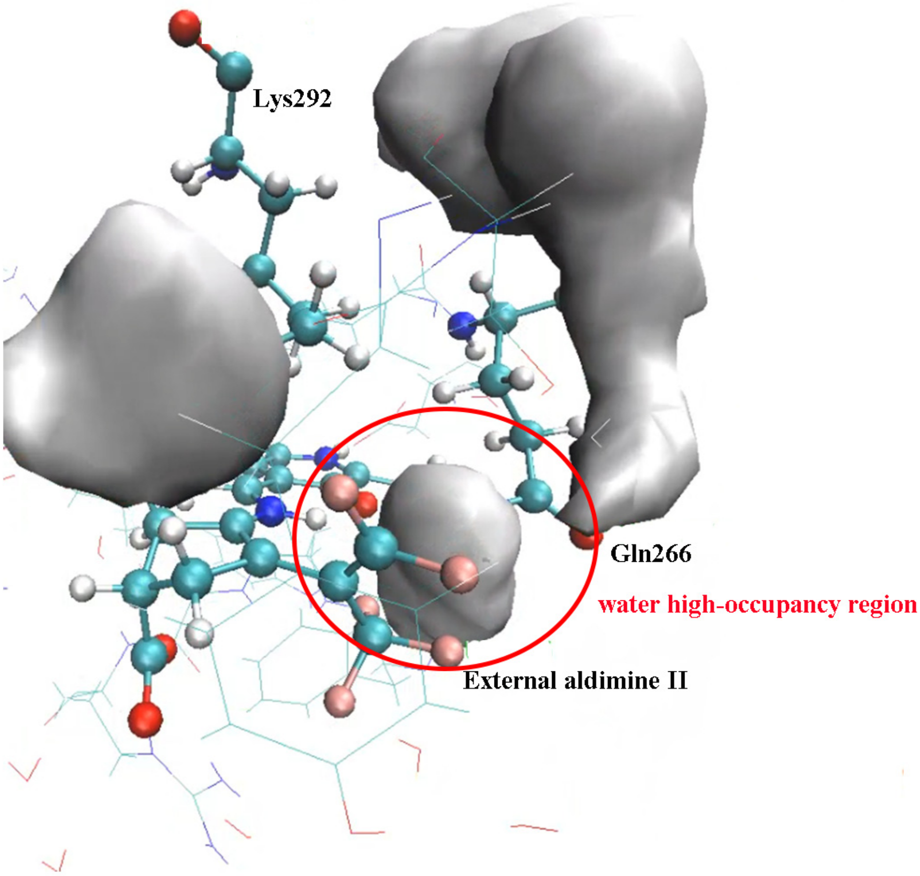

Figure S3. Fractional-occupancy volumetric map for water oxygen atoms from equilibrated 50 ns SBMD simulation. grey isosurface corresponds to occupancy ⩾ 0.7, marking the persistent hydration hot-spot in the interstitial cavity among Lys292, external aldimine II.

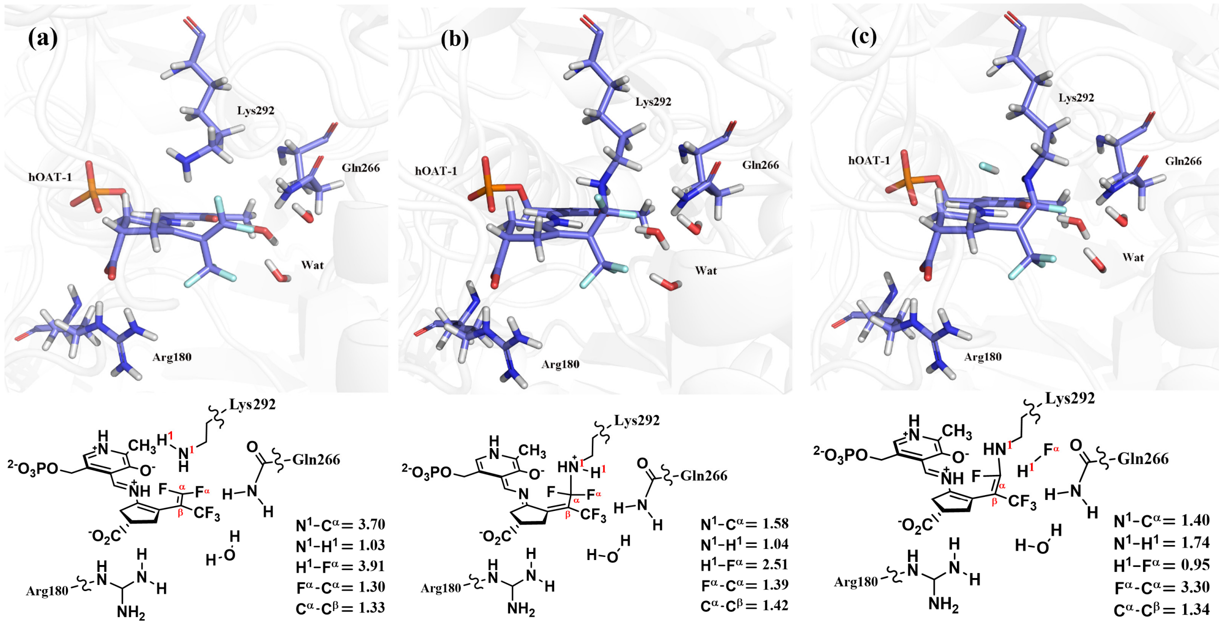

Figure S4. Optimized structures and key bond distances (32.56 ns) for the reactant (a) R, (b) IM1, (c) IM2.

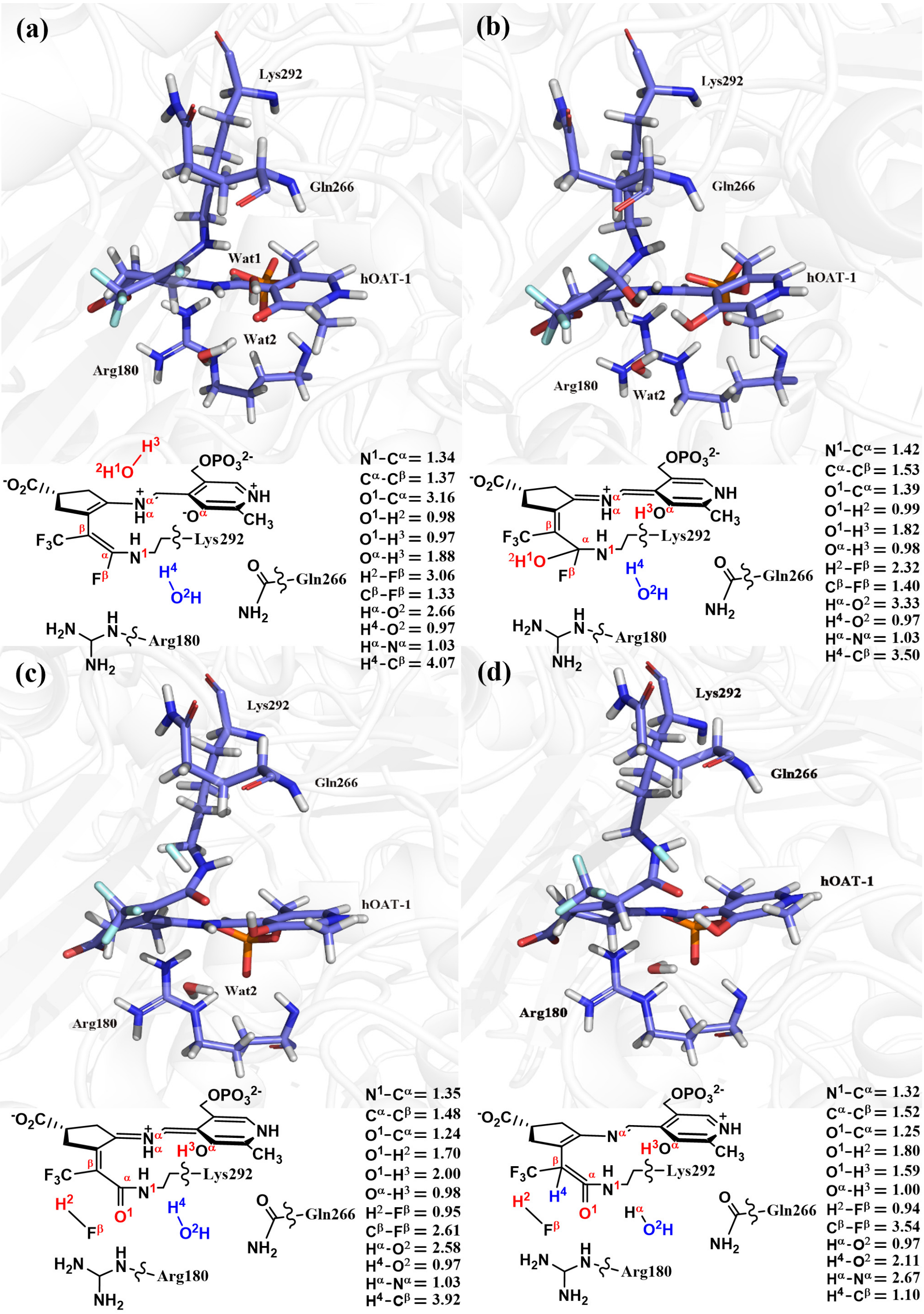

Figure S5. Optimized structures and key bond distances (32.56 ns) for the reactant (a) IM2', (b) IM3, (c) IM4, (d) P.

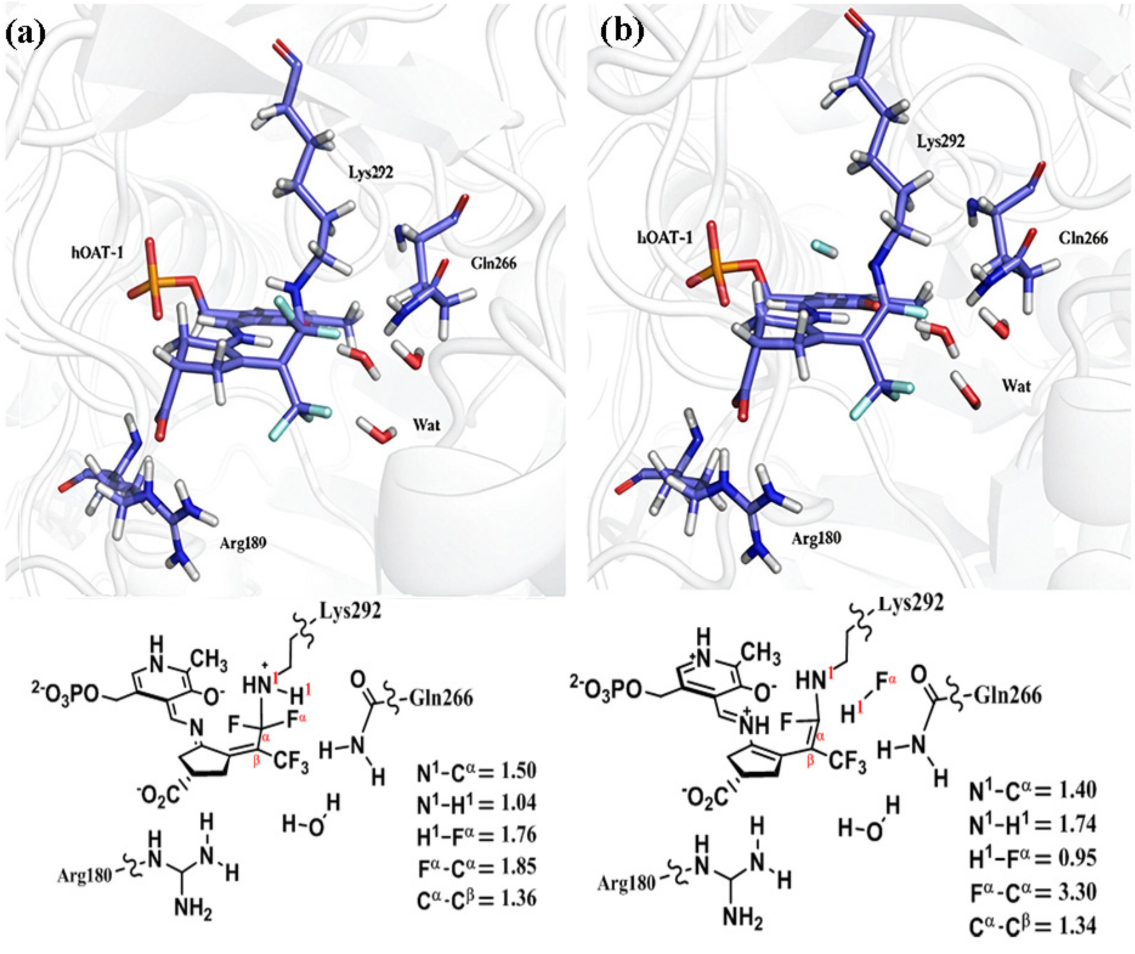

Figure S6. Structures and key bond distances of unoptimized pathway (32.56 ns) for the reactant (a) TS2', (b) IM2.

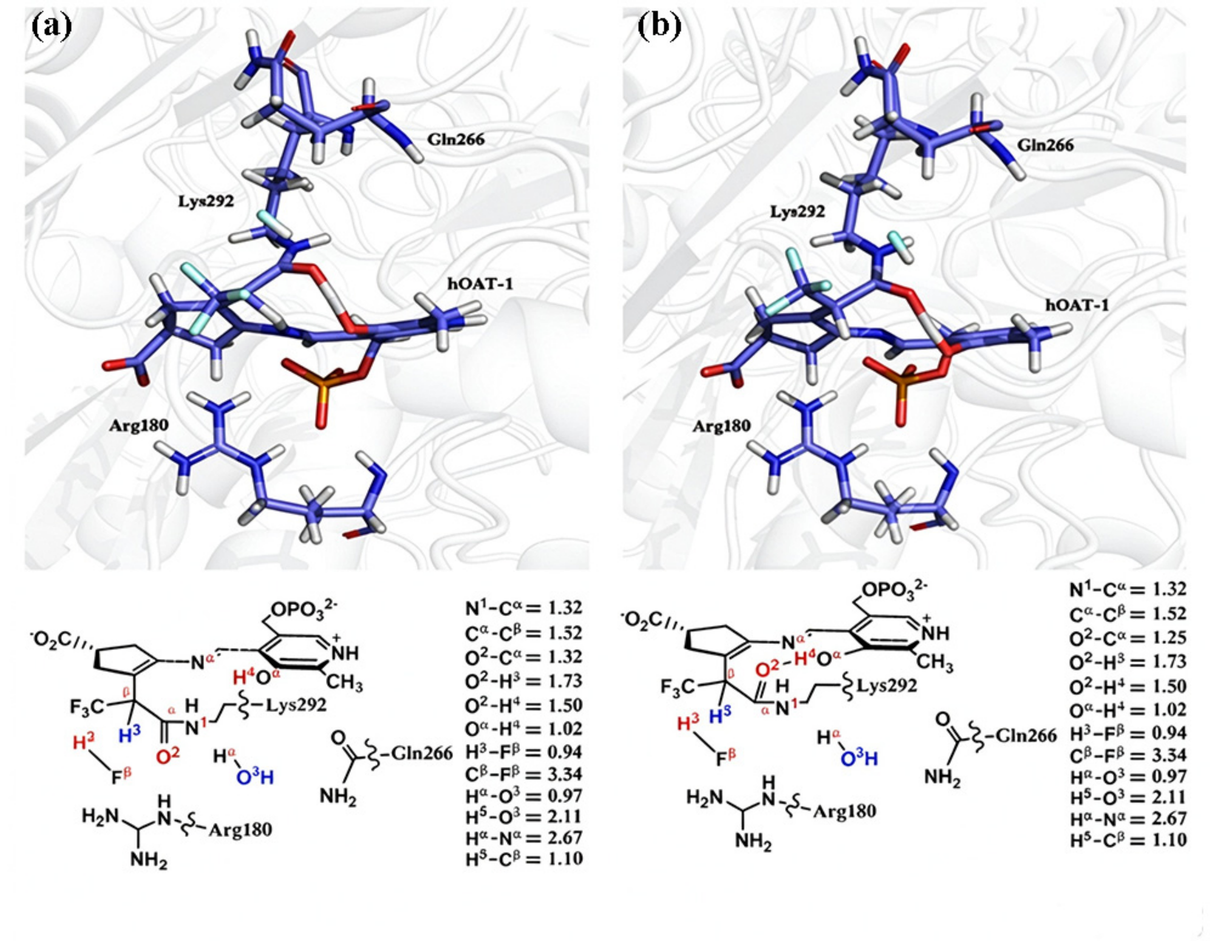

Figure S7. Structures and key bond distances of unoptimized pathway (32.56 ns) for the reactant (a) TS5’, (b) P’.

The force field of external aldimine II and Lss generated by SwissParam.

```
RESI SUB          -2.000 ! param penalty= 347.500 ; charge penalty= 250.965
GROUP            ! CHARGE   CH_PENALTY
ATOM N1     NG2R61 -0.200 !   51.166
ATOM C1     CG2R62 -0.219 !   52.654
ATOM C2     CG331  -0.168 !   32.166
ATOM C3     CG2R62  0.543 !   27.219
ATOM O1     OG312  -0.758 !   10.864
ATOM C4     CG2R62 -0.143 !   20.843
ATOM C5     CG2DC1  0.179 !   28.101
ATOM C6     CG2R62  0.040 !   17.654
ATOM C7     CG2R62  0.147 !    8.338
ATOM C8     CG321  -0.146 !   11.415
ATOM O2     OG303  -0.404 !   12.379
ATOM P      PG2     1.095 !    2.500
ATOM O3     OG2P1  -0.900 !    0.000
ATOM O4     OG2P1  -0.900 !    0.000
ATOM O5     OG2P1  -0.900 !    0.000
ATOM C9     CG302   0.455 !   81.983
ATOM C10    CG3C52 -0.193 !    9.008
ATOM C11    CG3C51 -0.091 !   38.164
ATOM C12    CG2DC1  0.391 !  246.076! typer WARNING: more "ne" statements than
neighbors
ATOM C13    CG3C52 -0.193 !    9.397
ATOM C14    CG2R51  0.096 !   94.431
ATOM C15    CG2R51  0.280 !  250.965
ATOM N2     NG2P1  -0.296 !  250.216
ATOM C16    CG2O3   0.519 !   38.115
ATOM C17    CG2DC1 -0.075 !  116.435! typer WARNING: more "ne" statements than
neighbors
ATOM F1     FGA3   -0.159 !   20.485
ATOM F2     FGA3   -0.159 !   20.485
ATOM F3     FGA3   -0.159 !   20.485
ATOM O6     OG2D2  -0.760 !    5.987
ATOM O7     OG2D2  -0.760 !    5.987
ATOM F4     FGA2   -0.188 !  174.084
ATOM F5     FGA2   -0.188 !  174.084
ATOM H1     HGA3    0.090 !    2.425
ATOM H2     HGA3    0.090 !    2.425
ATOM H3     HGA3    0.090 !    2.425
ATOM H4     HGR63   0.232 !    1.071
ATOM H5     HGA2    0.090 !    0.390
ATOM H6     HGA2    0.090 !    0.390
ATOM H7     HGA2    0.090 !    2.528
ATOM H8     HGA2    0.090 !    2.528
ATOM H9     HGA1    0.090 !    3.567
```

ATOM H10 HGA2 0.090 ! 2.528
ATOM H11 HGA2 0.090 ! 2.528
ATOM H12 HGP2 0.422 ! 2.500
ATOM H13 HGP2 0.460 ! 21.090
ATOM H14 HGR52 0.200 ! 2.710

BOND N1 C1
BOND N1 C7
BOND N1 H12
BOND C1 C3
BOND C1 C2
BOND C2 H1
BOND C2 H2
BOND C2 H3
BOND C3 O1
BOND C3 C4
BOND C4 C5
BOND C4 C6
BOND C5 N2
BOND C5 H14
BOND C6 C8
BOND C6 C7
BOND C7 H4
BOND C8 H6
BOND C8 H5
BOND C10 H8
BOND C10 C11
BOND C10 C15
BOND C10 H7
BOND C11 C13
BOND C11 H9
BOND C12 F4
BOND C12 F5
BOND C12 C17
BOND C13 H11
BOND C13 H10
BOND C13 C14
BOND C14 C17
BOND C14 C15
BOND C15 N2
BOND N2 H13
BOND C9 F1
BOND C9 C17
BOND C9 F2
BOND C9 F3
BOND C11 C16

BOND C16 O6
BOND C16 O7
BOND C8 O2
BOND P O4
BOND P O3
BOND O2 P
BOND P O5
IMPR C5 C4 N2 H14
IMPR C16 O7 O6 C11

BONDS
CG2DC1 CG2DC1 440.00 1.3400 ! RETINOL BTE2, 2-butene
CG2DC1 CG2R51 365.00 1.4500 ! lig , from CG2DC1 CG2R61, PENALTY= 45
CG2DC1 CG2R62 365.00 1.4500 ! lig , from CG2DC2 CG2R61, PENALTY= 5
CG2DC1 CG302 365.00 1.5020 ! lig , from CG2DC2 CG321, PENALTY= 55
CG2DC1 NG2P1 470.00 1.2830 ! RETINOL SCH2, Schiff's base, protonated
CG2DC1 FGA2 400.00 1.3580 ! lig , from CG2R66 FGR1, PENALTY= 145.5
CG2DC1 HGR52 360.50 1.1000 ! RETINOL SCH2, Schiff's base, protonated
CG2O3 CG3C51 250.00 1.4900 ! PROT 6-31g* AcProNH2, ProNH2, AcProNHCH3 RLD 4/23/93
CG2O3 OG2D2 525.00 1.2600 ! PROT adm jr. 7/23/91, acetic acid
CG2R51 CG2R51 410.00 1.3600 ! PROT histidine, adm jr., 6/27/90
CG2R51 CG3C52 350.00 1.5100 ! 2PRP, 2-pyrroline.H+; 2PRL, 2-pyrroline; 3PRL, 3-pyrroline, kevo
CG2R51 NG2P1 463.00 1.3650 ! lig , from CG2N1 NG2P1, PENALTY= 175
CG2R62 CG2R62 420.00 1.3500 ! NA nad/ppi, jjp1/adm jr. 7/95
CG2R62 CG321 230.00 1.4900 ! lig , from CG2R61 CG321, PENALTY= 5
CG2R62 CG331 230.00 1.4780 ! NA T, adm jr. 11/97
CG2R62 NG2R61 302.00 1.3430 ! NA C, adm jr. 11/97
CG2R62 OG312 525.00 1.2600 ! lig , from CG2R61 OG312, PENALTY= 5
CG2R62 HGR63 350.00 1.0900 ! NA nad/ppi, jjp1/adm jr. 7/95
CG302 FGA3 265.00 1.3400 ! FLUROALK fluoroalkanes
CG321 OG303 320.00 1.4400 ! PROTNA serine/threonine phosphate
CG321 HGA2 309.00 1.1110 ! PROT alkane update, adm jr., 3/2/92
CG331 HGA3 322.00 1.1110 ! PROT alkane update, adm jr., 3/2/92
CG3C51 CG3C52 195.00 1.5180 ! THF, nucleotide CSD/NDB survey, 5/30/06,viv
CG3C51 HGA1 307.00 1.1000 ! THF, THF neutron diffr., 5/30/06, viv
CG3C52 HGA2 307.00 1.1000 ! THF, THF neutron diffr., 5/30/06, viv
NG2P1 HGP2 455.00 1.0000 ! RETINOL SCH2, Schiff's base, protonated
NG2R61 HGP2 474.00 1.0100 ! NA C,U, JWK
OG2P1 PG2 400.00 1.5200 ! MP_2 reorganization, kevo
OG303 PG2 150.00 1.6550 ! MP_2 reorganization, kevo

ANGLES
CG2DC1 CG2DC1 CG2R51 29.00 122.00 ! lig , from CG2DC3 CG2DC1 CG2R61, PENALTY= 12

CG2DC1 CG2DC1 CG302 48.00 123.50 ! lig , from CG2DC1 CG2DC1 CG321, PENALTY= 12
CG2DC1 CG2DC1 FGA2 48.00 123.50 ! lig , from CG2DC1 CG2DC1 CG321, PENALTY= 43
CG2R51 CG2DC1 CG302 48.00 113.00 ! lig , from CG2DC2 CG2DC1 CG331, PENALTY= 35.4
CG2R62 CG2DC1 NG2P1 40.00 125.60 ! lig , from CG2DC2 CG2DC1 NG2P1, PENALTY= 22.5
CG2R62 CG2DC1 HGR52 32.00 120.00 ! lig , from CG2R61 CG2DC2 HGA4, PENALTY= 8.5
NG2P1 CG2DC1 HGR52 38.00 114.00 ! RETINOL SCH2, Schiff's base, protonated
FGA2 CG2DC1 FGA2 48.00 123.50 ! lig , from CG321 CG2DC1 CG331, PENALTY= 86.9
CG3C51 CG2O3 OG2D2 40.00 116.00 50.00 2.35300 ! PROT AcProNH2, ProNH2, AcProNHCH3 RLD 4/23/93
OG2D2 CG2O3 OG2D2 100.00 128.00 70.00 2.25870 ! PROT adm jr. 7/23/91, correction, ACETATE (KK)
CG2DC1 CG2R51 CG2R51 35.00 130.00 ! lig , from CG2O1 CG2R51 CG2R51, PENALTY= 29
CG2DC1 CG2R51 CG3C52 65.00 126.70 ! lig , from CG3C52 CG2R53 OG2D1, PENALTY= 83
CG2R51 CG2R51 CG3C52 115.00 109.00 ! 2PRP, 2-pyrroline.H+; 2PRL, 2-pyrroline, kevo
CG2R51 CG2R51 NG2P1 35.00 130.00 ! lig , from CG2O1 CG2R51 CG2R51, PENALTY= 53
CG3C52 CG2R51 NG2P1 65.00 126.70 ! lig , from CG3C52 CG2R53 OG2D1, PENALTY= 64
CG2DC1 CG2R62 CG2R62 36.00 120.00 ! lig , from CG2DC2 CG2R61 CG2R61, PENALTY= 5.5
CG2R62 CG2R62 CG2R62 40.00 118.00 ! NA nad/ppi, jjp1/adm jr. 7/95
CG2R62 CG2R62 CG321 40.00 124.20 ! lig , from CG2R62 CG2R62 CG331, PENALTY= 0.9
CG2R62 CG2R62 CG331 40.00 124.20 ! NA 5mc, adm jr. 9/9/93
CG2R62 CG2R62 NG2R61 85.00 122.90 ! NA C
CG2R62 CG2R62 OG312 40.00 120.00 ! lig , from CG2R61 CG2R61 OG312, PENALTY= 5.5
CG2R62 CG2R62 HGR63 80.00 120.50 ! NA nad/ppi, jjp1/adm jr. 7/95
CG331 CG2R62 NG2R61 45.80 122.30 ! lig , from CG331 CG2R61 NG2R60, PENALTY= 27
NG2R61 CG2R62 HGR63 80.00 117.50 ! NA nad/ppi, jjp1/adm jr. 7/95
CG2DC1 CG302 FGA3 50.00 115.00 30.00 2.35700 ! lig , from CG2R61 CG312 FGA2, PENALTY= 27.1
FGA3 CG302 FGA3 118.00 107.00 30.00 2.15500 ! FLUROALK fluoroalkanes
CG2R62 CG321 OG303 75.70 110.10 ! lig , from CG2R61 CG321 OG302, PENALTY= 3
CG2R62 CG321 HGA2 49.30 107.50 ! lig , from CG2R61 CG321 HGA2, PENALTY= 0.5
OG303 CG321 HGA2 60.00 109.50 ! PROTNA Thr-Phos

HGA2   CG321 HGA2   35.50   109.00   5.40   1.80200 ! PROT alkane update, adm jr., 3/2/92
CG2R62 CG331 HGA3   33.43   110.10   22.53   2.17900 ! NA Alkanes, sacred
HGA3   CG331 HGA3   35.50   108.40   5.40   1.80200 ! PROT alkane update, adm jr., 3/2/92
CG2O3 CG3C51 CG3C52   52.00   112.30 ! PROT AcProNH2, ProNH2, AcProNHCH3 RLD 4/23/93
CG2O3 CG3C51 HGA1   50.00   112.00 ! PROT AcProNH2, ProNH2, AcProNHCH3 RLD 4/23/93
CG3C52 CG3C51 CG3C52   58.00   109.50   11.16   2.56100 ! THF, nucleotide CSD/NDB survey, 05/30/06, viv
CG3C52 CG3C51 HGA1   35.00   111.40   22.53   2.17900 ! TF2M, viv
CG2R51 CG3C52 CG3C51   52.00   106.00 ! lig , from CG2R51 CG3C52 CG3C52, PENALTY= 0.4
CG2R51 CG3C52 HGA2   52.00   112.60 ! 2PRP, 2-pyrroline.H+; 2PRL, 2-pyrroline, kevo
CG3C51 CG3C52 HGA2   35.00   111.40   22.53   2.17900 ! TF2M, viv
HGA2   CG3C52 HGA2   38.50   106.80   5.40   1.80200 ! THF, 10/17/05 viv
CG2DC1 NG2P1 CG2R51   67.00   123.60 ! lig , from CG2DC1 NG2P1 CG334, PENALTY= 74.9
CG2DC1 NG2P1 HGP2   38.00   118.80 ! RETINOL SCH2, Schiff's base, protonated
CG2R51 NG2P1 HGP2   49.00   120.00 ! lig , from CG2N1 NG2P1 HGP2, PENALTY= 33
CG2R62 NG2R61 CG2R62   30.00   120.00 ! NA nad/ppi, jjp1/adm jr. 7/95
CG2R62 NG2R61 HGP2   32.00   117.40 ! NA nad/ppi, jjp1/adm jr. 7/95
CG321 OG303 PG2   20.00   120.00   35.00   2.33000 ! NA !Reorganization: TH5P and others
OG2P1 PG2   OG2P1   104.00   121.00 ! MP_2 reorganization, kevo
OG2P1 PG2   OG303   88.90   111.00 ! MP_2 reorganization, kevo

DIHEDRALS
CG2R51 CG2DC1 CG2DC1 FGA2   0.5600 1   180.00 ! lig , from CG2DC2 CG2DC1 CG2DC1 CG321, PENALTY= 65.5
CG2R51 CG2DC1 CG2DC1 FGA2   7.0000 2   180.00 ! lig , from CG2DC2 CG2DC1 CG2DC1 CG321, PENALTY= 65.5
CG302 CG2DC1 CG2DC1 FGA2   10.0000 2   180.00 ! lig , from CG321 CG2DC1 CG2DC1 CG331, PENALTY= 55.9
CG2DC1 CG2DC1 CG2R51 CG2R51   0.7500 2   180.00 ! lig , from CG2DC3 CG2DC1 CG2R61 CG2R61, PENALTY= 97
CG2DC1 CG2DC1 CG2R51 CG2R51   0.1900 4   0.00 ! lig , from CG2DC3 CG2DC1 CG2R61 CG2R61, PENALTY= 97
CG2DC1 CG2DC1 CG2R51 CG3C52   0.7500 2   180.00 ! lig , from CG2DC3 CG2DC1 CG2R61 CG2R61, PENALTY= 142
CG2DC1 CG2DC1 CG2R51 CG3C52   0.1900 4   0.00 ! lig , from CG2DC3 CG2DC1 CG2R61 CG2R61, PENALTY= 142
CG302 CG2DC1 CG2R51 CG2R51   1.1000 2   180.00 ! lig , from NG2S1 CG2O1 CG2R51 CG2R51, PENALTY= 151

CG302 CG2DC1 CG2R51 CG2R51   0.3000 4   0.00 ! lig , from NG2S1 CG2O1 CG2R51 CG2R51, PENALTY= 151
CG302 CG2DC1 CG2R51 CG3C52   0.5000 2   180.00 ! lig , from CG321 CG2DC1 CG2O1 NG2S2, PENALTY= 242.5
CG302 CG2DC1 CG2R51 CG3C52   0.3500 3   180.00 ! lig , from CG321 CG2DC1 CG2O1 NG2S2, PENALTY= 242.5
CG302 CG2DC1 CG2R51 CG3C52   0.4000 6   0.00 ! lig , from CG321 CG2DC1 CG2O1 NG2S2, PENALTY= 242.5
NG2P1 CG2DC1 CG2R62 CG2R62   1.6000 2   180.00 ! lig , from NG2D1 CG2DC1 CG2R61 CG2R61, PENALTY= 31.5
HGR52 CG2DC1 CG2R62 CG2R62   0.6000 2   180.00 ! lig , from HGA4 CG2DC1 CG2R61 CG2R61, PENALTY= 13.5
CG2DC1 CG2DC1 CG302 FGA3   0.7000 3   0.00 ! lig , from CG2DC1 CG2DC1 CG321 OG3R60, PENALTY= 83.2
CG2R51 CG2DC1 CG302 FGA3   0.3000 3   0.00 ! lig , from CG2DC2 CG2DC1 CG301 CG321, PENALTY= 132.5
CG2R62 CG2DC1 NG2P1 CG2R51   7.0000 2   180.00 ! lig , from CG2DC2 CG2DC1 NG2P1 CG334, PENALTY= 97.4
CG2R62 CG2DC1 NG2P1 HGP2   5.0000 2   180.00 ! lig , from CG2DC2 CG2DC1 NG2P1 HGP2, PENALTY= 22.5
HGR52 CG2DC1 NG2P1 CG2R51   8.5000 2   180.00 ! lig , from HGR52 CG2DC1 NG2P1 CG334, PENALTY= 74.9
HGR52 CG2DC1 NG2P1 HGP2   5.0000 2   180.00 ! RETINOL SCH2, Schiff's base, protonated
OG2D2 CG2O3 CG3C51 CG3C52   0.1600 3   0.00 ! PROT AcProNH2, ProNH2, AcProNHCH3 RLD 4/23/93
OG2D2 CG2O3 CG3C51 HGA1   0.1600 3   0.00 ! PROT AcProNH2, ProNH2, AcProNHCH3 RLD 4/23/93
CG2DC1 CG2R51 CG2R51 CG3C52   0.0000 2   180.00 ! lig , from CG2O1 CG2R51 CG2R51 CG321, PENALTY= 59
CG2DC1 CG2R51 CG2R51 NG2P1   0.0000 2   180.00 ! lig , from CG2O1 CG2R51 CG2R51 CG321, PENALTY= 96
CG3C52 CG2R51 CG2R51 CG3C52   12.0000 2   180.00 ! 3PRL, 3-pyrroline, kevo
CG3C52 CG2R51 CG2R51 NG2P1   2.5000 2   180.00 ! lig , from CG321 CG2R51 CG2R51 NG2R52, PENALTY= 72
CG2DC1 CG2R51 CG3C52 CG3C51   0.0500 3   180.00 ! lig , from CG2R51 CG2R51 CG3C52 CG3C52, PENALTY= 62.9
CG2DC1 CG2R51 CG3C52 HGA2   0.0000 3   0.00 ! lig , from CG2R51 CG2R51 CG3C52 HGA2, PENALTY= 62.5
CG2R51 CG2R51 CG3C52 CG3C51   0.0500 3   180.00 ! lig , from CG2R51 CG2R51 CG3C52 CG3C52, PENALTY= 0.4
CG2R51 CG2R51 CG3C52 HGA2   0.0000 3   0.00 ! 2PRP, 2-pyrroline.H+; 2PRL, 2-pyrroline, kevo
NG2P1 CG2R51 CG3C52 CG3C51   0.0800 3   0.00 ! lig , from OG2D1 CG2R53 CG3C52 CG3C52, PENALTY= 64.4

NG2P1 CG2R51 CG3C52 HGA2   0.0000 3   0.00 ! lig , from OG2D1 CG2R53 CG3C52 HGA2, PENALTY= 64
CG2R51 CG2R51 NG2P1 CG2DC1   1.2000 2   180.00 ! lig , from CG2R61 CG2R61 NG2S1 CG2O1, PENALTY= 302.5
CG2R51 CG2R51 NG2P1 HGP2   2.0000 2   180.00 ! lig , from CG2R61 CG2N2 NG2P1 HGP2, PENALTY= 233.5
CG3C52 CG2R51 NG2P1 CG2DC1   1.2000 2   180.00 ! lig , from CG2R61 CG2R61 NG2S1 CG2O1, PENALTY= 347.5
CG3C52 CG2R51 NG2P1 HGP2   3.5000 2   180.00 ! lig , from CG331 CG2N2 NG2P1 HGP2, PENALTY= 215.9
CG2DC1 CG2R62 CG2R62 CG2R62   3.1000 2   180.00 ! lig , from CG2DC1 CG2R61 CG2R61 CG2R61, PENALTY= 10.5
CG2DC1 CG2R62 CG2R62 CG321   3.1000 2   180.00 ! lig , from CG2R61 CG2R61 CG2R61 CG321, PENALTY= 51
CG2DC1 CG2R62 CG2R62 OG312   3.1000 2   180.00 ! lig , from CG2R61 CG2R61 CG2R61 OG312, PENALTY= 51
CG2R62 CG2R62 CG2R62 CG2R62   6.0000 2   180.00 ! NA nad/ppi, jjp1/adm jr. 7/95
CG2R62 CG2R62 CG2R62 CG321   3.1000 2   180.00 ! lig , from CG2R61 CG2R61 CG2R61 CG321, PENALTY= 10.5
CG2R62 CG2R62 CG2R62 CG331   3.1000 2   180.00 ! lig , from CG2R61 CG2R61 CG2R61 CG331, PENALTY= 10.5
CG2R62 CG2R62 CG2R62 NG2R61   7.0000 2   180.00 ! NA nad/ppi, jjp1/adm jr. 7/95
CG2R62 CG2R62 CG2R62 OG312   3.1000 2   180.00 ! lig , from CG2R61 CG2R61 CG2R61 OG312, PENALTY= 10.5
CG2R62 CG2R62 CG2R62 HGR63   1.0000 2   180.00 ! NA bases
CG321 CG2R62 CG2R62 NG2R61   4.0000 2   180.00 ! lig , from CG331 CG2R62 CG2R62 NG2R61, PENALTY= 0.9
CG321 CG2R62 CG2R62 HGR63   4.0000 2   180.00 ! lig , from CG331 CG2R62 CG2R62 HGR62, PENALTY= 1.9
CG331 CG2R62 CG2R62 OG312   2.4000 2   180.00 ! lig , from CG331 CG2R61 CG2R61 OG311, PENALTY= 25
NG2R61 CG2R62 CG2R62 OG312   1.0000 2   180.00 ! lig , from NG2R60 CG2R61 CG2R61 OG311, PENALTY= 47
CG2R62 CG2R62 CG321 OG303   0.0000 2   0.00 ! lig , from CG2R61 CG2R61 CG321 OG302, PENALTY= 8
CG2R62 CG2R62 CG321 HGA2   0.0020 6   0.00 ! lig , from CG2R61 CG2R61 CG321 HGA2, PENALTY= 5.5
CG2R62 CG2R62 CG331 HGA3   0.4600 3   0.00 ! NA T
NG2R61 CG2R62 CG331 HGA3   0.0030 6   180.00 ! lig , from NG2R60 CG2R61 CG331 HGA3, PENALTY= 27
CG2R62 CG2R62 NG2R61 CG2R62   4.0000 2   180.00 ! NA nad/ppi, jjp1/adm jr. 7/95
CG2R62 CG2R62 NG2R61 HGP2   1.0000 2   180.00 ! NA base
CG331 CG2R62 NG2R61 CG2R62   4.0000 2   180.00 ! lig , from NG2S3 CG2R64 NG2R61 CG2R63, PENALTY= 88.5
CG331 CG2R62 NG2R61 HGP2   0.0000 2   180.00 ! lig , from NG2S3 CG2R64 NG2R61 HGP1, PENALTY= 89.5

HGR63 CG2R62 NG2R61 CG2R62   7.0000 2   180.00 ! NA nad/ppi, jjp1/adm jr. 7/95
HGR63 CG2R62 NG2R61 HGP2   3.0000 2   180.00 ! NA nad/ppi, jjp1/adm jr. 7/95
CG2R62 CG321 OG303 PG2   0.6000 1   180.00 ! lig , from CG331 CG321 OG303 PG2, PENALTY= 71.9
CG2R62 CG321 OG303 PG2   0.6500 2   0.00 ! lig , from CG331 CG321 OG303 PG2, PENALTY= 71.9
CG2R62 CG321 OG303 PG2   0.0500 3   0.00 ! lig , from CG331 CG321 OG303 PG2, PENALTY= 71.9
HGA2   CG321 OG303 PG2   0.0000 3   0.00 ! NA dmp !Reorganization: TH5P and others
CG2O3 CG3C51 CG3C52 CG2R51   0.1400 3   0.00 ! lig , from CG2O3 CG3C51 CG3C52 CG3C52, PENALTY= 74
CG2O3 CG3C51 CG3C52 HGA2   0.1400 3   0.00 ! PROT AcProNH2, ProNH2, AcProNHCH3 RLD 4/23/93
CG3C52 CG3C51 CG3C52 CG2R51   3.7000 3   180.00 ! lig , from CG2R51 CG3C52 CG3C52 CG3C52, PENALTY= 4
CG3C52 CG3C51 CG3C52 CG2R51   2.2500 4   0.00 ! lig , from CG2R51 CG3C52 CG3C52 CG3C52, PENALTY= 4
CG3C52 CG3C51 CG3C52 CG2R51   0.2300 6   180.00 ! lig , from CG2R51 CG3C52 CG3C52 CG3C52, PENALTY= 4
CG3C52 CG3C51 CG3C52 HGA2   0.1900 3   0.00 ! alkane, 4/98, yin and mackerell, thf, viv
HGA1   CG3C51 CG3C52 CG2R51   0.1400 3   0.00 ! lig , from CG2R51 CG3C52 CG3C52 HGA2, PENALTY= 4
HGA1   CG3C51 CG3C52 HGA2   0.1900 3   0.00 ! alkane, 4/98, yin and mackerell, thf, viv
CG321 OG303 PG2   OG2P1   0.1000 3   0.00 ! NA dmp !Reorganization: TH5P and others

IMPROPERS
CG2DC1 CG2R62 NG2P1 HGR52   30.0000 0   0.00 ! lig , from CG2DC1 CG2R61 NG2D1 HGA4, PENALTY= 6.513
CG2O3 OG2D2 OG2D2 CG3C51   96.0000 0   0.00 ! lig , from CG2O3 OG2D2 OG2D2 CG321, PENALTY= 6.5

RESI LSS   -2.000 ! param penalty= 347.500 ; charge penalty= 250.965
GROUP   ! CHARGE   CH_PENALTY
ATOM N3   NG2R61 -0.200   !   48.226
ATOM C7   CG2R62 -0.219   !   48.430
ATOM C8   CG331 -0.168   !   31.267
ATOM C9   CG2R62 0.543   !   25.311
ATOM O2   OG311 -0.758   !   6.351
ATOM C10   CG2R62 -0.143   !   30.483
ATOM C11   CG2DC1 0.179   !   36.356
ATOM C12   CG2R62 0.040   !   18.492
ATOM C13   CG2R62 0.147   !   8.338
ATOM C14   CG321 -0.146   !   11.415

ATOM O3 OG303 -0.404 ! 12.379
ATOM P1 PG2 1.095 ! 2.500
ATOM O4 OG2P1 -0.900 ! 0.000
ATOM O5 OG2P1 -0.900 ! 0.000
ATOM O6 OG2P1 -0.900 ! 0.000
ATOM C15 CG302 0.455 ! 83.191
ATOM C16 CG3C52 -0.193 ! 9.008
ATOM C17 CG3C51 -0.091 ! 38.164
ATOM C18 CG2D1O 0.391 ! 177.533
ATOM C19 CG3C52 -0.193 ! 9.397
ATOM C20 CG2R51 0.096 ! 97.134
ATOM C21 CG2R51 0.280 ! 250.965
ATOM N4 NG2P1 -0.296 ! 250.216
ATOM C22 CG2O3 0.519 ! 38.115
ATOM C23 CG2DC1 -0.075 ! 118.098! typer WARNING: more "ne" statements than neighbors
ATOM F1 FGA3 -0.159 ! 20.485
ATOM F2 FGA3 -0.159 ! 20.485
ATOM F3 FGA3 -0.159 ! 20.485
ATOM O7 OG2D2 -0.710 ! 5.987
ATOM O8 OG2D2 -0.710 ! 5.987
ATOM F5 FGA1 -0.188 ! 177.258
ATOM H12 HGA3 0.095 ! 2.425
ATOM H13 HGA3 0.095 ! 2.425
ATOM H14 HGA3 0.095 ! 2.425
ATOM H15 HGR63 0.242 ! 1.071
ATOM H16 HGA2 0.095 ! 0.390
ATOM H17 HGA2 0.090 ! 0.390
ATOM H18 HGA2 0.090 ! 2.528
ATOM H19 HGA2 0.090 ! 2.528
ATOM H20 HGA1 0.090 ! 3.567
ATOM H21 HGA2 0.090 ! 2.528
ATOM H22 HGA2 0.090 ! 2.528
ATOM H23 HGP2 0.442 ! 2.500
ATOM H24 HGP2 0.462 ! 21.090
ATOM H25 HGR52 0.200 ! 2.710
!!!!!LYS
ATOM N NH1 -0.47 ! 19.229
ATOM HN H 0.31 ! 12.042
ATOM CA CT1 0.07 ! 13.708
ATOM HA HB1 0.09 ! 0.660
ATOM CB CT2 -0.18 ! 5.173
ATOM HB1 HA2 0.09 ! 0.418
ATOM HB2 HA2 0.09 ! 0.418
ATOM CG CT2 -0.18 ! 2.870
ATOM HG1 HA2 0.09 ! 0.000

```
ATOM HG2    HA2     0.09                  !    0.000
ATOM CD     CT2    -0.18                  !    2.525
ATOM HD1    HA2     0.09                  !    0.030
ATOM HD2    HA2     0.09                  !    0.030
ATOM CE     CT2     0.13                  !   15.422
ATOM HE1    HA2     0.075                 !    1.050
ATOM HE2    HA2     0.075                 !    1.050
ATOM NZ     NH2    -0.96                  !   41.088
ATOM HZ2    HC      0.34                  !    2.873
ATOM C      C       0.51                  !   16.562
ATOM O      O      -0.51                  !   14.582


BOND CA   N
BOND N    HN
BOND CA   CB
BOND C    +N
BOND CA   C
BOND CA   HA
BOND CB   HB1
BOND CB   HB2
BOND CB   CG
BOND CG   HG2
BOND CG   HG1
BOND CG   CD
BOND CD   CE
BOND CD   HD1
BOND CD   HD2
BOND CE   HE1
BOND CE   NZ
BOND CE   HE2
BOND NZ   HZ2
BOND NZ   C18
BOND N3   C13
BOND N3   C7
BOND N3   H23
BOND C7   C8
BOND C7   C9
BOND C8   H13
BOND C8   H14
BOND C8   H12
BOND C9   O2
BOND C9   C10
BOND C10  C11
BOND C10  C12
BOND C11  H25
```

```
BOND C11  N4
BOND C12  C13
BOND C12  C14
BOND C13  H15
BOND C14  O3
BOND C14  H16
BOND C14  H17
BOND C15  F2
BOND C15  C23
BOND C15  F3
BOND C15  F1
BOND C16  C21
BOND C16  H18
BOND C16  H19
BOND C16  C17
BOND C17  H20
BOND C17  C19
BOND C17  C22
BOND C18  C23
BOND C18  F5
BOND C19  H21
BOND C19  C20
BOND C19  H22
BOND C20  C23
BOND C20  C21
BOND C21  N4
BOND N4   H24
BOND C22  O8
BOND C22  O7
BOND O3   P1
BOND P1   O5
BOND P1   O6
BOND P1   O4
DOUBLE   O  C
IMPR N -C CA HN  C CA +N O
CMAP -C  N  CA  C   N  CA  C  +N
IMPR C11    C10    N4     H25
IMPR C18    C23    NZ     F5
IMPR C22    O7     O8     C17
DONOR HN N
ACCEPTOR O C
IC -C   CA   *N   HN    1.3482 123.5700  180.0000 115.1100  0.9988
IC -C   N    CA   C     1.3482 123.5700  180.0000 107.2900  1.5187
IC N    CA   C    +N    1.4504 107.2900  180.0000 117.2700  1.3478
IC +N   CA   *C   O     1.3478 117.2700  180.0000 120.7900  1.2277
IC CA   C    +N   +CA   1.5187 117.2700  180.0000 124.9100  1.4487
```

IC N C *CA CB 1.4504 107.2900 122.2300 111.3600 1.5568
IC N C *CA HA 1.4504 107.2900 -116.8800 107.3600 1.0833
IC N CA CB CG 1.4504 111.4700 180.0000 115.7600 1.5435
IC CG CA *CB HB1 1.5435 115.7600 120.9000 107.1100 1.1146
IC CG CA *CB HB2 1.5435 115.7600 -124.4800 108.9900 1.1131
IC CA CB CG CD 1.5568 115.7600 180.0000 113.2800 1.5397
IC CD CB *CG HG1 1.5397 113.2800 120.7400 109.1000 1.1138
IC CD CB *CG HG2 1.5397 113.2800 -122.3400 108.9900 1.1143
IC CB CG CD CE 1.5435 113.2800 180.0000 112.3300 1.5350
IC CE CG *CD HD1 1.5350 112.3300 122.2500 108.4100 1.1141
IC CE CG *CD HD2 1.5350 112.3300 -121.5900 108.1300 1.1146
IC CG CD CE NZ 1.5397 112.3300 180.0000 110.4600 1.4604
IC NZ CD *CE HE1 1.4604 110.4600 119.9100 110.5100 1.1128
IC NZ CD *CE HE2 1.4604 110.4600 -120.0200 110.5700 1.1123
IC C18 CE *NZ HZ2 1.0404 110.0200 120.2700 109.5000 1.0402

BONDS
CG2D1O CG2DC1 440.00 1.3400 ! PY02, 2h-pyran
CG2D1O NH2 420.00 1.3550 ! NICH; Kenno: reverted to nadh/ppi, jjp1/adm jr. 7/95
CG2D1O FGA1 400.00 1.3580 ! lig , from CG2R66 FGR1, PENALTY= 126.6
CG2DC1 CG2R51 365.00 1.4500 ! lig , from CG2DC1 CG2R61, PENALTY= 45
CG2DC1 CG2R62 365.00 1.4500 ! lig , from CG2DC2 CG2R61, PENALTY= 5
CG2DC1 CG302 365.00 1.5020 ! lig , from CG2DC2 CG321, PENALTY= 55
CG2DC1 NG2P1 470.00 1.2830 ! RETINOL SCH2, Schiff's base, protonated
CG2DC1 HGR52 360.50 1.1000 ! RETINOL SCH2, Schiff's base, protonated
CG2O3 CG3C51 250.00 1.4900 ! PROT 6-31g* AcProNH2, ProNH2, AcProNHCH3 RLD 4/23/93
CG2O3 OG2D2 525.00 1.2600 ! PROT adm jr. 7/23/91, acetic acid
CG2O4 CG311 250.00 1.5000 ! lig , from CG2O4 CG321, PENALTY= 4
CG2O4 OG2D1 700.00 1.2150 ! ALDEHYDE acetaldehyde adm 11/08
CG2O4 HGR52 330.00 1.1100 ! ALDEHYDE acetaldehyde adm 11/08
CG2R51 CG2R51 410.00 1.3600 ! PROT histidine, adm jr., 6/27/90
CG2R51 CG3C52 350.00 1.5100 ! 2PRP, 2-pyrroline.H+; 2PRL, 2-pyrroline; 3PRL, 3-pyrroline, kevo
CG2R51 NG2P1 463.00 1.3650 ! lig , from CG2N1 NG2P1, PENALTY= 175
CG2R62 CG2R62 420.00 1.3500 ! NA nad/ppi, jjp1/adm jr. 7/95
CG2R62 CG321 230.00 1.4900 ! lig , from CG2R61 CG321, PENALTY= 5
CG2R62 CG331 230.00 1.4780 ! NA T, adm jr. 11/97
CG2R62 NG2R61 302.00 1.3430 ! NA C, adm jr. 11/97
CG2R62 OG311 334.30 1.4110 ! lig , from CG2R61 OG311, PENALTY= 5
CG2R62 HGR63 350.00 1.0900 ! NA nad/ppi, jjp1/adm jr. 7/95
CG302 FGA3 265.00 1.3400 ! FLUROALK fluoroalkanes
CG311 CG321 222.50 1.5380 ! PROT alkane update, adm jr., 3/2/92
CG311 NG321 263.00 1.4740 ! lig , from CG321 NG321, PENALTY= 4
CG311 HGA1 309.00 1.1110 ! PROT alkane update, adm jr., 3/2/92
CG321 CG321 222.50 1.5300 ! PROT alkane update, adm jr., 3/2/92

CG321 OG303   320.00   1.4400 ! PROTNA serine/threonine phosphate
CG321 HGA2   309.00   1.1110 ! PROT alkane update, adm jr., 3/2/92
CG331 HGA3   322.00   1.1110 ! PROT alkane update, adm jr., 3/2/92
CG3C51 CG3C52 195.00   1.5180 ! THF, nucleotide CSD/NDB survey, 5/30/06,viv
CG3C51 HGA1   307.00   1.1000 ! THF, THF neutron diffr., 5/30/06, viv
CG3C52 HGA2   307.00   1.1000 ! THF, THF neutron diffr., 5/30/06, viv
NG2P1 HGP2   455.00   1.0000 ! RETINOL SCH2, Schiff's base, protonated
NG2R61 HGP2   474.00   1.0100 ! NA C,U, JWK
NG321 HGPAM2 453.10   1.0140 ! AMINE aliphatic amines
OG2P1 PG2   400.00   1.5200 ! MP_2 reorganization, kevo
OG303 PG2   150.00   1.6550 ! MP_2 reorganization, kevo
OG311 HGP1   545.00   0.9600 ! PROT EMB 11/21/89 methanol vib fit; og tested on MeOH EtOH,...

CG2DC1 CG2D1O NH2   60.00   122.00 ! NICH; Kenno: reverted to nadh/ppi, jjp1/adm jr. 7/95
CG2DC1 CG2D1O FGA1   56.00   124.50 ! lig , from CG2DC1 CG2D1O OG301, PENALTY= 25
NH2   CG2D1O FGA1   48.00   123.50 ! lig , from CG321 CG2D1 CG331, PENALTY= 112.9
CG2D1O CG2DC1 CG2R51   29.00   122.00 ! lig , from CG2DC3 CG2DC1 CG2R61, PENALTY= 14
CG2D1O CG2DC1 CG302   43.50   126.50 ! lig , from CG2D1O CG2DC1 CG321, PENALTY= 12
CG2R51 CG2DC1 CG302   48.00   113.00 ! lig , from CG2DC2 CG2DC1 CG331, PENALTY= 35.4
CG2R62 CG2DC1 NG2P1   40.00   125.60 ! lig , from CG2DC2 CG2DC1 NG2P1, PENALTY= 22.5
CG2R62 CG2DC1 HGR52   32.00   120.00 ! lig , from CG2R61 CG2DC2 HGA4, PENALTY= 8.5
NG2P1 CG2DC1 HGR52   38.00   114.00 ! RETINOL SCH2, Schiff's base, protonated
CG3C51 CG2O3 OG2D2   40.00   116.00   50.00   2.35300 ! PROT AcProNH2, ProNH2, AcProNHCH3 RLD 4/23/93
OG2D2 CG2O3 OG2D2   100.00   128.00   70.00   2.25870 ! PROT adm jr. 7/23/91, correction, ACETATE (KK)
CG311 CG2O4 OG2D1   45.00   126.00 ! lig , from CG321 CG2O4 OG2D1, PENALTY= 0.6
CG311 CG2O4 HGR52   65.00   116.00 ! lig , from CG321 CG2O4 HGR52, PENALTY= 0.6
OG2D1 CG2O4 HGR52   65.00   118.00 ! ALDEHYDE acetaldehyde adm 11/08
CG2DC1 CG2R51 CG2R51   35.00   130.00 ! lig , from CG2O1 CG2R51 CG2R51, PENALTY= 29
CG2DC1 CG2R51 CG3C52   65.00   126.70 ! lig , from CG3C52 CG2R53 OG2D1, PENALTY= 83
CG2R51 CG2R51 CG3C52 115.00   109.00 ! 2PRP, 2-pyrroline.H+; 2PRL, 2-pyrroline, kevo
CG2R51 CG2R51 NG2P1   35.00   130.00 ! lig , from CG2O1 CG2R51 CG2R51, PENALTY= 53

CG3C52 CG2R51 NG2P1 65.00 126.70 ! lig , from CG3C52 CG2R53 OG2D1, PENALTY= 64
CG2DC1 CG2R62 CG2R62 36.00 120.00 ! lig , from CG2DC2 CG2R61 CG2R61, PENALTY= 5.5
CG2R62 CG2R62 CG2R62 40.00 118.00 ! NA nad/ppi, jjp1/adm jr. 7/95
CG2R62 CG2R62 CG321 40.00 124.20 ! lig , from CG2R62 CG2R62 CG331, PENALTY= 0.9
CG2R62 CG2R62 CG331 40.00 124.20 ! NA 5mc, adm jr. 9/9/93
CG2R62 CG2R62 NG2R61 85.00 122.90 ! NA C
CG2R62 CG2R62 OG311 45.20 120.00 ! lig , from CG2R61 CG2R61 OG311, PENALTY= 5.5
CG2R62 CG2R62 CG2DC1 40.00 124.20
CG2DC1 CG2R62 CG2R62 40.00 124.20
CG2R62 CG2R62 HGR63 80.00 120.50 ! NA nad/ppi, jjp1/adm jr. 7/95
CG331 CG2R62 NG2R61 45.80 122.30 ! lig , from CG331 CG2R61 NG2R60, PENALTY= 27
NG2R61 CG2R62 CG331 45.80 122.30 ! lig , from CG331 CG2R61 NG2R60, PENALTY= 27
NG2R61 CG2R62 HGR63 80.00 117.50 ! NA nad/ppi, jjp1/adm jr. 7/95
CG2DC1 CG302 FGA3 50.00 115.00 30.00 2.35700 ! lig , from CG2R61 CG312 FGA2, PENALTY= 27.1
FGA3 CG302 FGA3 118.00 107.00 30.00 2.15500 ! FLUROALK fluoroalkanes
CG2O4 CG311 CG321 52.00 108.00 ! lig , from CG2O2 CG311 CG321, PENALTY= 2
CG2O4 CG311 NG321 43.70 110.00 ! lig , from CG2O2 CG321 NG321, PENALTY= 6
CG2O4 CG311 HGA1 50.00 109.50 ! lig , from CG2O5 CG311 HGA1, PENALTY= 0.5
CG321 CG311 NG321 43.70 110.00 ! lig , from CG321 CG321 NH2, PENALTY= 5.2
CG321 CG311 HGA1 34.50 110.10 22.53 2.17900 ! PROT alkane update, adm jr., 3/2/92
NG321 CG311 HGA1 32.40 109.50 50.00 2.14000 ! lig , from NG321 CG321 HGA2, PENALTY= 4
CG2R62 CG321 OG303 75.70 110.10 ! lig , from CG2R61 CG321 OG302, PENALTY= 3
CG2R62 CG321 HGA2 49.30 107.50 ! lig , from CG2R61 CG321 HGA2, PENALTY= 0.5
CG311 CG321 CG321 58.35 113.50 11.16 2.56100 ! PROT alkanes
CG311 CG321 HGA2 33.43 110.10 22.53 2.17900 ! PROT alkanes
CG321 CG321 CG321 58.35 113.60 11.16 2.56100 ! PROT alkane update, adm jr., 3/2/92
CG321 CG321 HGA2 26.50 110.10 22.53 2.17900 ! PROT alkane update, adm jr., 3/2/92
OG303 CG321 HGA2 60.00 109.50 ! PROTNA Thr-Phos
HGA2 CG321 HGA2 35.50 109.00 5.40 1.80200 ! PROT alkane update, adm jr., 3/2/92
CG2R62 CG331 HGA3 33.43 110.10 22.53 2.17900 ! NA Alkanes, sacred
HGA3 CG331 HGA3 35.50 108.40 5.40 1.80200 ! PROT alkane update, adm jr., 3/2/92
CG2O3 CG3C51 CG3C52 52.00 112.30 ! PROT AcProNH2, ProNH2, AcProNHCH3 RLD 4/23/93

CG2O3 CG3C51 HGA1   50.00   112.00 ! PROT AcProNH2, ProNH2, AcProNHCH3 RLD 4/23/93
CG3C52 CG3C51 CG3C52   58.00   109.50   11.16   2.56100 ! THF, nucleotide CSD/NDB survey, 05/30/06, viv
CG3C52 CG3C51 HGA1   35.00   111.40   22.53   2.17900 ! TF2M, viv
CG2R51 CG3C52 CG3C51   52.00   106.00 ! lig , from CG2R51 CG3C52 CG3C52, PENALTY= 0.4
CG2R51 CG3C52 HGA2   52.00   112.60 ! 2PRP, 2-pyrroline.H+; 2PRL, 2-pyrroline, kevo
CG3C51 CG3C52 HGA2   35.00   111.40   22.53   2.17900 ! TF2M, viv
HGA2   CG3C52 HGA2   38.50   106.80   5.40   1.80200 ! THF, 10/17/05 viv
CG2DC1 NG2P1 CG2R51   67.00   123.60 ! lig , from CG2DC1 NG2P1 CG334, PENALTY= 74.9
CG2DC1 NG2P1 HGP2   38.00   118.80 ! RETINOL SCH2, Schiff's base, protonated
CG2R51 NG2P1 HGP2   49.00   120.00 ! lig , from CG2N1 NG2P1 HGP2, PENALTY= 33
CG2R62 NG2R61 CG2R62   30.00   120.00 ! NA nad/ppi, jjp1/adm jr. 7/95
CG2R62 NG2R61 HGP2   32.00   117.40 ! NA nad/ppi, jjp1/adm jr. 7/95
CG2D1O NH2   CT2   70.00   121.70 ! lig , from CG2D2O NG301 CG3C51, PENALTY= 16.4
CT2   NH2   CG2D1O   70.00   121.70 ! lig , from CG2D2O NG301 CG3C51, PENALTY= 16.4
CG2D1O NH2   HC   39.00   123.00 ! NICH; Kenno: reverted to nad/ppi, jjp1/adm jr. 7/95
HC   NH2   CG2D1O   39.00   123.00 ! NICH; Kenno: reverted to nad/ppi, jjp1/adm jr. 7/95
CG311 NG321 HGPAM2   41.00   112.10 ! lig , from CG321 NG321 HGPAM2, PENALTY= 0.6
HGPAM2 NG321 HGPAM2   42.00   105.85 ! AMINE aliphatic amines kevo: 29.50 -> 42.00 based on MAM1 molvib & AMET scans
CG321 OG303 PG2   20.00   120.00   35.00   2.33000 ! NA !Reorganization: TH5P and others
CG2R62 OG311 HGP1   65.00   108.00 ! lig , from CG2R61 OG311 HGP1, PENALTY= 0.5
OG2P1 PG2   OG2P1   104.00   121.00 ! MP_2 reorganization, kevo
OG2P1 PG2   OG303   88.90   111.00 ! MP_2 reorganization, kevo

DIHEDRALS
NH2   CG2D1O CG2DC1 CG2R51   2.5000 2   180.00 ! lig , from NH2 CG2D1O CG2DC1 CG2O1, PENALTY= 30
NH2   CG2D1O CG2DC1 CG302   2.5000 2   180.00 ! lig , from NH2 CG2D1O CG2DC1 CG321, PENALTY= 12
FGA1   CG2D1O CG2DC1 CG2R51   1.5000 1   180.00 ! lig , from OG301 CG2D1O CG2DC1 CG2DC2, PENALTY= 47.5
FGA1   CG2D1O CG2DC1 CG2R51   15.0000 2   180.00 ! lig , from OG301 CG2D1O CG2DC1 CG2DC2, PENALTY= 47.5
FGA1   CG2D1O CG2DC1 CG302   2.5000 2   180.00 ! lig , from NH2 CG2D1O CG2DC1 CG321, PENALTY= 46
CG2DC1 CG2D1O NH2   CT2   0.1000 2   180.00 ! lig , from CG2DC1 CG2D1O NG301 CG3C51, PENALTY= 16.4

CG2DC1 CG2D1O NH2 HC 0.1000 2 180.00 ! NICH; Kenno: reverted to nad/ppi, jjp1/adm jr. 7/95
FGA1 CG2D1O NH2 CT2 0.1000 2 180.00 ! lig , from CG2D1 CG2D1O NG301 CG3C51, PENALTY= 115.4
FGA1 CG2D1O NH2 HC 0.1000 2 180.00 ! lig , from CG2D1 CG2D1O NH2 HC, PENALTY= 99
CG2D1O CG2DC1 CG2R51 CG2R51 0.7500 2 180.00 ! lig , from CG2DC3 CG2DC1 CG2R61 CG2R61, PENALTY= 99
CG2D1O CG2DC1 CG2R51 CG2R51 0.1900 4 0.00 ! lig , from CG2DC3 CG2DC1 CG2R61 CG2R61, PENALTY= 99
CG2D1O CG2DC1 CG2R51 CG3C52 0.7500 2 180.00 ! lig , from CG2DC3 CG2DC1 CG2R61 CG2R61, PENALTY= 144
CG2D1O CG2DC1 CG2R51 CG3C52 0.1900 4 0.00 ! lig , from CG2DC3 CG2DC1 CG2R61 CG2R61, PENALTY= 144
CG302 CG2DC1 CG2R51 CG2R51 1.1000 2 180.00 ! lig , from NG2S1 CG2O1 CG2R51 CG2R51, PENALTY= 151
CG302 CG2DC1 CG2R51 CG2R51 0.3000 4 0.00 ! lig , from NG2S1 CG2O1 CG2R51 CG2R51, PENALTY= 151
CG302 CG2DC1 CG2R51 CG3C52 0.5000 2 180.00 ! lig , from CG321 CG2DC1 CG2O1 NG2S2, PENALTY= 242.5
CG302 CG2DC1 CG2R51 CG3C52 0.3500 3 180.00 ! lig , from CG321 CG2DC1 CG2O1 NG2S2, PENALTY= 242.5
CG302 CG2DC1 CG2R51 CG3C52 0.4000 6 0.00 ! lig , from CG321 CG2DC1 CG2O1 NG2S2, PENALTY= 242.5
NG2P1 CG2DC1 CG2R62 CG2R62 1.6000 2 180.00 ! lig , from NG2D1 CG2DC1 CG2R61 CG2R61, PENALTY= 31.5
HGR52 CG2DC1 CG2R62 CG2R62 0.6000 2 180.00 ! lig , from HGA4 CG2DC1 CG2R61 CG2R61, PENALTY= 13.5
CG2D1O CG2DC1 CG302 FGA3 0.7000 3 0.00 ! lig , from CG2DC1 CG2DC1 CG321 OG3R60, PENALTY= 86.7
CG2R51 CG2DC1 CG302 FGA3 0.3000 3 0.00 ! lig , from CG2DC2 CG2DC1 CG301 CG321, PENALTY= 132.5
CG2R62 CG2DC1 NG2P1 CG2R51 7.0000 2 180.00 ! lig , from CG2DC2 CG2DC1 NG2P1 CG334, PENALTY= 97.4
CG2R62 CG2DC1 NG2P1 HGP2 5.0000 2 180.00 ! lig , from CG2DC2 CG2DC1 NG2P1 HGP2, PENALTY= 22.5
HGR52 CG2DC1 NG2P1 CG2R51 8.5000 2 180.00 ! lig , from HGR52 CG2DC1 NG2P1 CG334, PENALTY= 74.9
HGR52 CG2DC1 NG2P1 HGP2 5.0000 2 180.00 ! RETINOL SCH2, Schiff's base, protonated
OG2D2 CG2O3 CG3C51 CG3C52 0.1600 3 0.00 ! PROT AcProNH2, ProNH2, AcProNHCH3 RLD 4/23/93
OG2D2 CG2O3 CG3C51 HGA1 0.1600 3 0.00 ! PROT AcProNH2, ProNH2, AcProNHCH3 RLD 4/23/93
OG2D1 CG2O4 CG311 CG321 1.0500 1 180.00 ! lig , from OG2D1 CG2O4 CG321 CG331, PENALTY= 4.9

OG2D1 CG2O4 CG311 CG321 0.4000 2 180.00 ! lig , from OG2D1 CG2O4 CG321 CG331, PENALTY= 4.9
OG2D1 CG2O4 CG311 CG321 0.6000 3 180.00 ! lig , from OG2D1 CG2O4 CG321 CG331, PENALTY= 4.9
OG2D1 CG2O4 CG311 CG321 0.1000 4 180.00 ! lig , from OG2D1 CG2O4 CG321 CG331, PENALTY= 4.9
OG2D1 CG2O4 CG311 NG321 0.0000 6 180.00 ! lig , from OG2D1 CG2O2 CG321 NG321, PENALTY= 34
OG2D1 CG2O4 CG311 HGA1 0.0000 3 180.00 ! lig , from OG2D1 CG2O4 CG321 HGA2, PENALTY= 4
HGR52 CG2O4 CG311 CG321 0.0000 3 180.00 ! lig , from HGR52 CG2O4 CG321 CG331, PENALTY= 4.9
HGR52 CG2O4 CG311 NG321 0.0000 3 180.00 ! lig , from HGR52 CG2O4 CG321 CG331, PENALTY= 43.9
HGR52 CG2O4 CG311 HGA1 0.0000 3 180.00 ! lig , from HGR52 CG2O4 CG321 HGA2, PENALTY= 4
CG2DC1 CG2R51 CG2R51 CG3C52 0.0000 2 180.00 ! lig , from CG2O1 CG2R51 CG2R51 CG321, PENALTY= 59
CG2DC1 CG2R51 CG2R51 NG2P1 0.0000 2 180.00 ! lig , from CG2O1 CG2R51 CG2R51 CG321, PENALTY= 96
CG3C52 CG2R51 CG2R51 CG3C52 12.0000 2 180.00 ! 3PRL, 3-pyrroline, kevo
CG3C52 CG2R51 CG2R51 NG2P1 2.5000 2 180.00 ! lig , from CG321 CG2R51 CG2R51 NG2R52, PENALTY= 72
CG2DC1 CG2R51 CG3C52 CG3C51 0.0500 3 180.00 ! lig , from CG2R51 CG2R51 CG3C52 CG3C52, PENALTY= 62.9
CG2DC1 CG2R51 CG3C52 HGA2 0.0000 3 0.00 ! lig , from CG2R51 CG2R51 CG3C52 HGA2, PENALTY= 62.5
CG2R51 CG2R51 CG3C52 CG3C51 0.0500 3 180.00 ! lig , from CG2R51 CG2R51 CG3C52 CG3C52, PENALTY= 0.4
CG2R51 CG2R51 CG3C52 HGA2 0.0000 3 0.00 ! 2PRP, 2-pyrroline.H+; 2PRL, 2-pyrroline, kevo
NG2P1 CG2R51 CG3C52 CG3C51 0.0800 3 0.00 ! lig , from OG2D1 CG2R53 CG3C52 CG3C52, PENALTY= 64.4
NG2P1 CG2R51 CG3C52 HGA2 0.0000 3 0.00 ! lig , from OG2D1 CG2R53 CG3C52 HGA2, PENALTY= 64
CG2R51 CG2R51 NG2P1 CG2DC1 1.2000 2 180.00 ! lig , from CG2R61 CG2R61 NG2S1 CG2O1, PENALTY= 302.5
CG2R51 CG2R51 NG2P1 HGP2 2.0000 2 180.00 ! lig , from CG2R61 CG2N2 NG2P1 HGP2, PENALTY= 233.5
CG3C52 CG2R51 NG2P1 CG2DC1 1.2000 2 180.00 ! lig , from CG2R61 CG2R61 NG2S1 CG2O1, PENALTY= 347.5
CG3C52 CG2R51 NG2P1 HGP2 3.5000 2 180.00 ! lig , from CG331 CG2N2 NG2P1 HGP2, PENALTY= 215.9
CG2DC1 CG2R62 CG2R62 CG2R62 3.1000 2 180.00 ! lig , from CG2DC1 CG2R61 CG2R61 CG2R61, PENALTY= 10.5

CG2DC1 CG2R62 CG2R62 CG321 3.1000 2 180.00 ! lig , from CG2R61 CG2R61 CG2R61 CG321, PENALTY= 51
CG2DC1 CG2R62 CG2R62 OG311 3.9000 2 180.00 ! lig , from CG2R61 CG2R62 CG2R62 OG3R60, PENALTY= 49.2
CG2R62 CG2R62 CG2R62 CG2R62 6.0000 2 180.00 ! NA nad/ppi, jjp1/adm jr. 7/95
CG2R62 CG2R62 CG2R62 CG321 3.1000 2 180.00 ! lig , from CG2R61 CG2R61 CG2R61 CG321, PENALTY= 10.5
CG2R62 CG2R62 CG2R62 CG331 3.1000 2 180.00 ! lig , from CG2R61 CG2R61 CG2R61 CG331, PENALTY= 10.5
CG2R62 CG2R62 CG2R62 NG2R61 7.0000 2 180.00 ! NA nad/ppi, jjp1/adm jr. 7/95
CG2R62 CG2R62 CG2R62 OG311 0.4000 2 180.00 ! lig , from CG2R62 CG2R62 CG2R62 OG3R60, PENALTY= 8.2
CG2R62 CG2R62 CG2R62 HGR63 1.0000 2 180.00 ! NA bases
CG321 CG2R62 CG2R62 NG2R61 4.0000 2 180.00 ! lig , from CG331 CG2R62 CG2R62 NG2R61, PENALTY= 0.9
CG321 CG2R62 CG2R62 HGR63 4.0000 2 180.00 ! lig , from CG331 CG2R62 CG2R62 HGR62, PENALTY= 1.9
CG331 CG2R62 CG2R62 OG311 2.4000 2 180.00 ! lig , from CG331 CG2R61 CG2R61 OG311, PENALTY= 10
NG2R61 CG2R62 CG2R62 OG311 1.0000 2 180.00 ! lig , from NG2R60 CG2R61 CG2R61 OG311, PENALTY= 32
CG2R62 CG2R62 CG321 OG303 0.0000 2 0.00 ! lig , from CG2R61 CG2R61 CG321 OG302, PENALTY= 8
CG2R62 CG2R62 CG321 HGA2 0.0020 6 0.00 ! lig , from CG2R61 CG2R61 CG321 HGA2, PENALTY= 5.5
CG2R62 CG2R62 CG331 HGA3 0.4600 3 0.00 ! NA T
NG2R61 CG2R62 CG331 HGA3 0.0030 6 180.00 ! lig , from NG2R60 CG2R61 CG331 HGA3, PENALTY= 27
CG2R62 CG2R62 NG2R61 CG2R62 4.0000 2 180.00 ! NA nad/ppi, jjp1/adm jr. 7/95
CG2R62 CG2R62 NG2R61 HGP2 1.0000 2 180.00 ! NA base
CG331 CG2R62 NG2R61 CG2R62 4.0000 2 180.00 ! lig , from NG2S3 CG2R64 NG2R61 CG2R63, PENALTY= 88.5
CG331 CG2R62 NG2R61 HGP2 0.0000 2 180.00 ! lig , from NG2S3 CG2R64 NG2R61 HGP1, PENALTY= 89.5
HGR63 CG2R62 NG2R61 CG2R62 7.0000 2 180.00 ! NA nad/ppi, jjp1/adm jr. 7/95
HGR63 CG2R62 NG2R61 HGP2 3.0000 2 180.00 ! NA nad/ppi, jjp1/adm jr. 7/95
CG2R62 CG2R62 OG311 HGP1 0.9900 2 180.00 ! lig , from CG2R61 CG2R61 OG311 HGP1, PENALTY= 5.5
CG2O4 CG311 CG321 CG321 0.2000 3 0.00 ! lig , from CG2O2 CG311 CG321 CG321, PENALTY= 2
CG2O4 CG311 CG321 HGA2 0.2000 3 0.00 ! lig , from CG2O2 CG311 CG321 HGA2, PENALTY= 2
NG321 CG311 CG321 CG321 0.1600 1 180.00 ! lig , from CG321 CG321 CG321 NG301, PENALTY= 5.8
NG321 CG311 CG321 CG321 0.3900 2 0.00 ! lig , from CG321 CG321 CG321 NG301, PENALTY= 5.8

NG321 CG311 CG321 HGA2 0.1950 3 0.00 ! lig , from NH2 CG321 CG321 HGA2, PENALTY= 5.2
HGA1 CG311 CG321 CG321 0.1950 3 0.00 ! NA abasic nucleoside
HGA1 CG311 CG321 HGA2 0.1950 3 0.00 ! NA, sugar
CG2O4 CG311 NG321 HGPAM2 0.1600 3 0.00 ! lig , from CG2O2 CG321 NG321 HGPAM2, PENALTY= 6
CG321 CG311 NG321 HGPAM2 0.3000 3 0.00 ! lig , from CG321 CG321 NH2 HC, PENALTY= 14.5
HGA1 CG311 NG321 HGPAM2 0.0100 3 0.00 ! lig , from HGA2 CG321 NG321 HGPAM2, PENALTY= 4
CG311 CG321 CG321 CG321 0.5000 3 0.00 ! CARBOCY carbocyclic sugars
CG311 CG321 CG321 CG321 0.5000 6 180.00 ! CARBOCY carbocyclic sugars
CG311 CG321 CG321 HGA2 0.1950 3 0.00 ! NA abasic nucleoside
CG321 CG321 CG321 CG321 0.0645 2 0.00 ! LIPID alkane, 4/04, jbk (Jeff Klauda)
CG321 CG321 CG321 CG321 0.1497 3 180.00 ! LIPID alkane, 4/04, jbk
CG321 CG321 CG321 CG321 0.0946 4 0.00 ! LIPID alkane, 4/04, jbk
CG321 CG321 CG321 CG321 0.1125 5 0.00 ! LIPID alkane, 4/04, jbk
CG321 CG321 CG321 HGA2 0.1950 3 0.00 ! LIPID alkanes
HGA2 CG321 CG321 HGA2 0.2200 3 0.00 ! LIPID alkanes
CT2 CT2 NH2 CG2D1O 2.5000 1 180.00 ! lig , from NH2 CG321 NH2 CG2R61, PENALTY= 60.5
CT2 CT2 NH2 CG2D1O 1.5000 2 0.00 ! lig , from NH2 CG321 NH2 CG2R61, PENALTY= 60.5
CT2 CT2 NH2 CG2D1O 0.5000 3 0.00 ! lig , from NH2 CG321 NH2 CG2R61, PENALTY= 60.5
HA2 CT2 NH2 CG2D1O 0.0000 3 180.00 ! lig , from HGA2 CG321 NH2 CG2R61, PENALTY= 21
HA2 CT2 NH2 HC 0.0500 3 0.00 ! PEI0, OBTZ, AOBT, kevo & xxwy
CG2R62 CG321 OG303 PG2 0.6000 1 180.00 ! lig , from CG331 CG321 OG303 PG2, PENALTY= 71.9
CG2R62 CG321 OG303 PG2 0.6500 2 0.00 ! lig , from CG331 CG321 OG303 PG2, PENALTY= 71.9
CG2R62 CG321 OG303 PG2 0.0500 3 0.00 ! lig , from CG331 CG321 OG303 PG2, PENALTY= 71.9
HGA2 CG321 OG303 PG2 0.0000 3 0.00 ! NA dmp !Reorganization: TH5P and others
CG2O3 CG3C51 CG3C52 CG2R51 0.1400 3 0.00 ! lig , from CG2O3 CG3C51 CG3C52 CG3C52, PENALTY= 74
CG2O3 CG3C51 CG3C52 HGA2 0.1400 3 0.00 ! PROT AcProNH2, ProNH2, AcProNHCH3 RLD 4/23/93
CG3C52 CG3C51 CG3C52 CG2R51 3.7000 3 180.00 ! lig , from CG2R51 CG3C52 CG3C52 CG3C52, PENALTY= 4
CG3C52 CG3C51 CG3C52 CG2R51 2.2500 4 0.00 ! lig , from CG2R51 CG3C52 CG3C52 CG3C52, PENALTY= 4
CG3C52 CG3C51 CG3C52 CG2R51 0.2300 6 180.00 ! lig , from CG2R51 CG3C52 CG3C52 CG3C52, PENALTY= 4

CG3C52 CG3C51 CG3C52 HGA2 0.1900 3 0.00 ! alkane, 4/98, yin and mackerell, thf, viv
HGA1 CG3C51 CG3C52 CG2R51 0.1400 3 0.00 ! lig , from CG2R51 CG3C52 CG3C52 HGA2, PENALTY= 4
HGA1 CG3C51 CG3C52 HGA2 0.1900 3 0.00 ! alkane, 4/98, yin and mackerell, thf, viv
CG321 OG303 PG2 OG2P1 0.1000 3 0.00 ! NA dmp !Reorganization: TH5P and others

IMPROPER
CG2D1O CG2DC1 NH2 FGA1 53.0000 0 0.00 ! lig , from CG2D1O CG2DC1 NH2 HGA4, PENALTY= 54.3
CG2DC1 CG2R62 NG2P1 HGR52 30.0000 0 0.00 ! lig , from CG2DC1 CG2R61 NG2D1 HGA4, PENALTY= 6.513
CG2O3 OG2D2 OG2D2 CG3C51 96.0000 0 0.00 ! lig , from CG2O3 OG2D2 OG2D2 CG321, PENALTY= 6.5
CG2O4 CG311 OG2D1 HGR52 50.0000 0 0.00 ! lig , from CG2O4 CG321 OG2D1 HGR52, PENALTY= 0.4

NONBONDED nbxmod 5 atom cdiel fshift vatom vdistance vfswitch -
cutnb 14.0 ctofnb 12.0 ctonnb 10.0 eps 1.0 e14fac 1.0 wmin 1.5
HGA1 0.0 -0.0450 1.3400 ! alkane, igor, 6/05
HGA2 0.0 -0.0350 1.3400 ! alkane, igor, 6/05
HGA3 0.0 -0.0240 1.3400 ! alkane, yin and mackerell, 4/98
HGA4 0.0 -0.0310 1.2500 ! alkene, yin,adm jr., 12/95
HGA5 0.0 -0.0260 1.2600 ! alkene, yin,adm jr., 12/95
HGA6 0.0 -0.0280 1.3200 ! fluoro_alkanes
HGA7 0.0 -0.0300 1.3000 ! fluoro_alkanes
HGAAM0 0.0 -0.0280 1.2800 ! aliphatic amines
HGAAM1 0.0 -0.0280 1.2800 ! aliphatic amines
HGAAM2 0.0 -0.0400 1.2600 ! aliphatic amines
HGP1 0.0 -0.0460 0.2245 ! polar H
HGP2 0.0 -0.0460 0.2245 ! small polar Hydrogen, charged systems
HGP3 0.0 -0.1000 0.4500 ! methanethiol pure solvent, adm jr., 6/22/92
HGP4 0.0 -0.0460 0.2245 ! polar H, conjugated amines (NA bases)
HGP5 0.0 -0.0460 0.7000 ! polar H on quarternary amine (choline)
HGPAM1 0.0 -0.0090 0.8750 ! aliphatic amines
HGPAM2 0.0 -0.0100 0.8750 ! aliphatic amines
HGPAM3 0.0 -0.0120 0.8700 ! aliphatic amines
HGR51 0.0 -0.0300 1.3582 ! benzene
HGR52 0.0 -0.0460 0.9000 ! adm jr., 6/27/90, his
HGR53 0.0 -0.0460 0.7000 ! adm jr., 6/27/90, his
HGR61 0.0 -0.0300 1.3582 ! benzene
HGR62 0.0 -0.0460 1.1000 ! intermediate aromatic Hvdw
HGR63 0.0 -0.0460 0.9000 ! nad/ppi, jjp1/adm jr.
HGR71 0.0 -0.0300 1.3582 ! benzene
